# Gene therapy for hereditary hearing loss by SLC26A4 mutations in mice reveals distinct functional roles of pendrin in normal hearing

**DOI:** 10.7150/thno.38032

**Published:** 2019-09-23

**Authors:** Min-A Kim, Sung Huhn Kim, Nari Ryu, Ji-Hyun Ma, Ye-Ri Kim, Jinsei Jung, Chuan-Jen Hsu, Jae Young Choi, Kyu-Yup Lee, Philine Wangemann, Jinwoong Bok, Un-Kyung Kim

**Affiliations:** 1 Department of Biology, College of Natural Sciences, Kyungpook National University, Daegu 41566, Republic of Korea; 2 School of Life Sciences, BK21 Plus KNU Creative BioResearch Group, Kyungpook National University, Daegu 41566, Republic of Korea; 3 Department of Otorhinolaryngology, Head and Neck Surgery, Yonsei University College of Medicine, Seoul 03722, Republic of Korea; 4 Department of Anatomy, Yonsei University College of Medicine, Seoul 03722, Republic of Korea; 5 BK21PLUS Project for Medical Science, Yonsei University College of Medicine, Seoul 03722, Republic of Korea; 6 Department of Otolaryngology, College of Medicine, National Taiwan University, Taipei, Taiwan; 7 Department of Otorhinolaryngology-Head and Neck Surgery, School of Medicine, Kyungpook National University, Daegu 41944, Republic of Korea; 8 Department of Anatomy and Physiology, Kansas State University, Manhattan, United States of America

**Keywords:** Solute carrier family 26 member 4, Enlarged vestibular aqueduct, Recombinant adeno-associated virus, Gene therapy, *In-utero*, Pendred syndrome

## Abstract

**Rationale**: Mutations of *SLC26A4* that abrogate pendrin, expressed in endolymphatic sac, cochlea and vestibule, are known to cause autosomal recessive sensorineural hearing loss with enlargement of the membranous labyrinth. This is the first study to demonstrate the feasibility of gene therapy for pendrin-related hearing loss.

**Methods:** We used a recombinant viral vector to transfect *Slc26a4* cDNA into embryonic day 12.5 otocysts of pendrin-deficient knock-out (*Slc26a4^∆/∆^*) and pendrin-deficient knock-in (*Slc26a4^tm1Dontuh/tm1Dontuh^*) mice.

**Results**: Local gene-delivery resulted in spatially and temporally limited pendrin expression, prevented enlargement, failed to restore vestibular function, but succeeded in the restoration of hearing. Restored hearing phenotypes included normal hearing as well as sudden, fluctuating, and progressive hearing loss.

**Conclusion**: Our study illustrates the feasibility of gene therapy for pendrin-related hearing loss, suggests differences in the requirement of pendrin between the cochlea and the vestibular labyrinth, and documents that insufficient pendrin expression during late embryonal and early postnatal development of the inner ear can cause sudden, fluctuating and progressive hearing loss without obligatory enlargement of the membranous labyrinth.

## Introduction

Mutations in the *SLC26A4* gene, which encodes the anion exchanger pendrin, are worldwide the second most prevalent cause of hereditary hearing loss; the most prevalent causes are mutations of *GJB2* 1. In some large populations, including those of East Asia, mutations of *SLC26A4* occur in 13-14% of patients with hereditary hearing loss 2, 3. Mutations of *SLC26A4* cause hearing loss associated with enlargement of the vestibular aqueduct (EVA), which can be non-syndromic (DFNB4, OMIM #600791) or syndromic with thyroid goiter (Pendred syndrome, OMIM #605646) 4, 5. Hearing loss can be profound at birth or fluctuating and progressive during childhood 6-8. Vestibular dysfunction is common but often well compensated 9.

**Figure 1:**
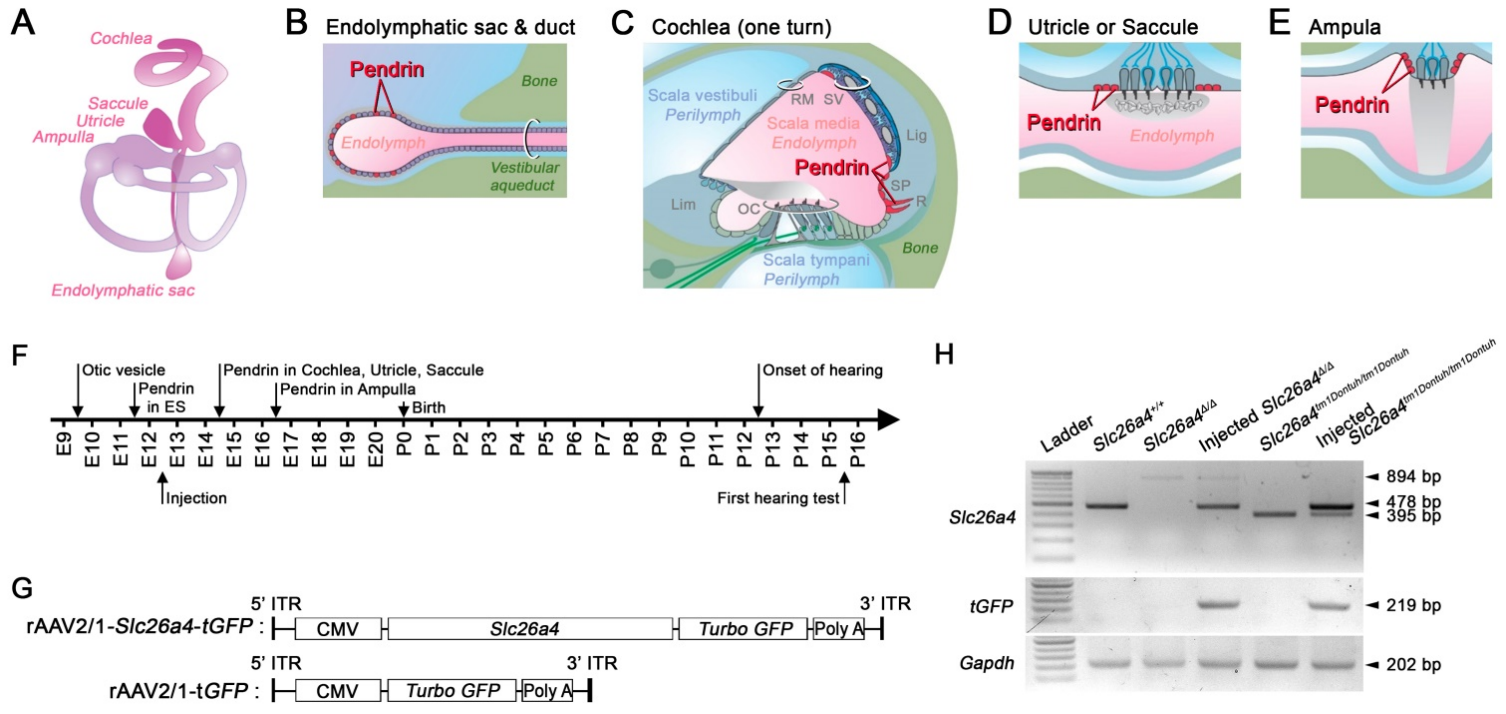
** Transcription of recombinant viral vectors in the embryonic inner ear.** (**A**) Schematic diagram of the membranous inner ear. (**B-E**) Schematic diagrams of parts of the inner ear where natural pendrin expression occurs. (**B**) Schematic cross-section of the endolymphatic sac and duct. (**C**) Schematic cross-section of one turn of the cochlea. Lig, spiral ligament; Lim, limbus; OC, organ of Corti; RM, Reissner's membrane; R, root cell; SP, spiral prominence; SV, stria vascularis. (**D**) Schematic cross-section of the utricle or saccule. (**E**) Schematic cross-section of an ampulla. (**F**) Onset of pendrin expression in different parts of the inner ear during development from embryonic (E) day 9 to postnatal (P) day 16. (**G**) Schematic diagrams of viral vectors. The rAAV2/1-*Slc26a4*-*tGFP* vector expressed *Slc26a4* and *turbo GFP* (*tGFP*) driven by the same CMV promoter. The rAAV2/1-*tGFP* vector, which was used as a control, expressed only tGFP under the control of the CMV promoter. CMV, cytomegalovirus; ITR, inverted terminal repeat from AAV2; Slc26a4, solute carrier family 26 member 4. GFP, green fluorescent protein. (**H**) mRNA expression in the inner ear. Transcripts of endogenous or vector-induced *Slc26a4^+^* mRNA (478 bp), of *Slc26a4^Δ^* (894 bp) and *Slc26a4^tm1Dontuh^* (395 bp) mRNA as well as vector-induced tGFP (219 bp) mRNA and endogenous *Gapdh* (202 bp) mRNA were amplified by RT-PCR and separated by gel-electrophoresis. Reactions were performed with total RNA isolated from inner ears of *Slc26a4^+/+^*, *Slc26a4^Δ/Δ^*, rAAV2/1-*Slc26a4*-*tGFP* injected *Slc26a4^Δ/Δ^*, *Slc26a4^tm1Dontuh/tm1Dontuh^*, and rAAV2/1-*Slc26a4*-*tGFP* injected *Slc26a4^tm1Dontuh/tm1Dontuh^* mice. The ladder consisted of markers between 100 and 1000 bp in 100 bp intervals.

The inner ear is comprised of the cochlea, vestibule, and endolymphatic sac (Figure 1A). The cochlea and vestibule detect mechanical stimulations such as sound waves and linear or angular accelerations and the endolymphatic sac regulates endolymph volume 10. Endolymph, the luminal fluid of the inner ear, has unique ionic compositions such as high [K^+^] and low [Na^+^] in the cochlea and vestibule and high [Na^+^] and low [K^+^] in the endolymphatic sac 11. The unique ionic milieu of endolymph, which is required for normal hearing and vestibular function, is regulated by various ion channels, transporters, and pumps expressed in inner ear epithelial and mesenchymal cells.

Pendrin is an exchanger for anions such as of Cl^-^ and HCO_3_^-^ that contributes to fluid homeostasis 12-15. Pendrin is expressed in epithelial cells in the inner ear including spindle cells of stria vascularis, outer sulcus and spiral prominence cells of the cochlea, transitional cells in the vestibular labyrinth, and mitochondria-rich cells of the endolymphatic sac (Figure 1A-E) 16, 17. During development of the murine inner ear, pendrin expression is first observed at embryonic day (E) 11.5 in the endolymphatic sac, where expression increases dramatically between E13.5 and E14.5 12, 18. Pendrin expression in the cochlea, saccule, and utricle begins at E14.5 and in the crista ampullaris at E16.5 (Figure 1F) 12.

The pathogenesis of hearing loss caused by *SLC26A4* mutations has been studied in mouse models, mainly in a knock-out *Slc26a4^∆/∆^*, a knock-in *Slc26a4^tm1Dontuh/tm1Dontuh^*, and an ENU-induced *Slc26a4^loop/loop^* mutant, and in a couple of transgenic models 19-23. Mouse models congenitally lacking functional pendrin exhibited profound hearing loss and vestibular dysfunction together with enlargement of endolymphatic spaces 19, 20. Studies in transgenic mouse models led to the hypothesis that temporally and spatially limited restoration of pendrin function is sufficient to prevent hearing loss in *Slc26a4*-deficient mice 21, 22. Choi *et al*. demonstrated that the requirement of pendrin expression is temporally limited to the seven day period from E16.5 to postnatal day (P) 2 21 and Li *et al*. reported that the requirement of pendrin expression is spatially limited to the endolymphatic sac 22.

We hypothesized that local gene delivery is suitable to restore hearing and balance in *Slc26a4*-deficient mice. We used a recombinant viral vector to deliver *Slc26a4* to otocysts of *Slc26a4*-deficient *Slc26a4^∆/∆^* and *Slc26a4*-deficient *Slc26a4^tm1Dontuh/tm1Dontuh^* mice. Gene-delivery resulted in spatially and temporally limited pendrin expression, prevented enlargement, failed to restore vestibular function, but succeeded to restore hearing. Restored hearing phenotypes included normal hearing as well as sudden, fluctuating, and progressive hearing loss. To our knowledge, local gene delivery has never before been used for *Slc26a4* or to target the endolymphatic sac of the inner ear. Our study illustrates the feasibility of gene therapy for pendrin-related hearing loss, suggests differences in the pendrin requirement for the cochlea and the vestibular labyrinth, and suggest that sudden, fluctuating and progressive hearing loss can be the result of insufficient pendrin expression even when no enlargement of the membranous labyrinth is observed.

## Materials and Methods

### Animals

*Slc26a4^∆/∆^* and *Slc26a4^tm1Dontuh/tm1Dontuh^* mice were obtained from the National Institutes of Health (USA) and the University of Taiwan, respectively 19, 20 and maintained free of known and suspected murine pathogens in a pathogen-free animal facility. *Slc26a4^∆/∆^* and *Slc26a4^+/+^* mice were generated by mating *Slc26a4^∆/+^* mice maintained in the 129S background. *Slc26a4^tm1Dontuh/tm1Dontuh^*and *Slc26a4^+/+^* mice were generated by mating *Slc26a4^tm1Dontuh/+^* mice maintained in the C57BL/6 background. All animal protocols were approved by the Institutional Animal Care and Use Committee of at the Kyungpook National University and Yonsei University College of Medicine.

### Local gene delivery via recombinant adeno-associated virus (rAAV)

Recombinant adeno-associated virus (rAAV) used in the present study contained the inverted terminal repeat (ITR) of AAV serotype 2, the capsid of AAV serotype 1 (rAAV2/1) and a cytomegalovirus (CMV) promoter to drive expression. rAAV2/1 were packaged either with murine *Slc26a4* cDNA (2,342 bp CDS excluding 3' and 5' UTR) fused with *turbo green fluorescent protein* (*tGFP*) gene (rAAV2/1-*Slc26a4*-*tGFP*) or with *tGFP* gene (rAAV2/1-*tGFP*) (Figure S1). Pre-packaged rAAVs were obtained from SignaGen Laboratories (Rockville, MD, USA). Titers of stocks, defined as vector genome copies per milliliter (VG/ml), were 1.08x10^13^ VG/ml for rAAV2/1-*Slc26a4* and 1.1x10^13^ VG/ml for rAAV2/1-*tGFP*. Approximately 0.6-1 µl of rAAV2/1-*Slc26a4-tGFP* or rAAV2/1-*tGFP* (1.08x10^10^ VG for rAAV2/1-*Slc26a4* and 1.1x10^10^ VG for rAAV2/1-*tGFP*) was microinjected into the left otocyst of E12.5 embryos. Stocks were stored at -80°C and thawed immediately prior to use.

Time-pregnant dams were generated by mating for 1 or 2 days. Pregnancy was assumed based on observation of a vaginal plug. The developmental age at the day a vaginal plug was observed was set to E0.5. Pregnant dams were anesthetized by a mixture of alfaxan (4 mg/100 g) and xylazine hydrochloride (0.13 mg/100 g) by intramuscular (i.m.) injection and were placed on a heating pad. All surgical procedures were conducted as described elsewhere 24.

### Histology and immunocytochemistry

#### Fixation

Mice were deeply anesthetized with a mixture of alfaxan (4 mg/100 g) and xylazine hydrochloride (0.13 mg/100 g) by i.m. injection. The postnatal mice were fixed by cardiac perfusion with 4% paraformaldehyde (PFA) in phosphate buffered saline (PBS), and then the inner ears were isolated from experimental mice. Extracted temporal bones, including the inner ear, from embryonic mice were fixed by submersion in 4% PFA in PBS without cardiac perfusion.

#### Cryosections

Fixed temporal bones from postnatal mice were decalcified for 24-48 hours (h) in 10% ethylenediaminetetraacetic acid (EDTA) in PBS. All tissues were dehydrated with 30% sucrose in PBS and a 1:1 30% sucrose:Optimal Cutting Temperature (OCT) compound mixture (Leica Microsystems, Bensheim, Germany) for 24 h each at 4°C. The temporal bones were then embedded in OCT compound. The embedded blocks were serially sectioned by cutting the tissues into 10-μm-thick slices using a microtome (Leica CM1850, Leica Microsystems, Bensheim, Germany).

#### Paraffin sections

The fixed inner ears were decalcified with 10% EDTA in PBS for 24 h at 4°C, dehydrated with a graded ethanol series, permeabilized with xylene, and embedded in paraffin at 65°C to initiate the embedding, and then cooled at room temperature (RT) to solidify the paraffin. The paraffin-embedded inner ears were then serially sectioned into 5-μm-thick slices using a microtome (Leica RM2235, Leica Microsystems, Bensheim, Germany). All tissue sections were mounted on Superfrost Plus microscope slides (Fisher Scientific, Pittsburgh, PA, USA). The slides with cryosections and paraffin sections were maintained at -80°C and RT, respectively, until use.

#### Immunofluorescence and direct GFP fluorescence

Immunofluorescence assays were conducted as described previously 24 with some modifications: primary antibodies were a rabbit polyclonal anti-pendrin antibody, that recognizes the amino acids CKDPLDLMEAEMNAEELDVQDEAMRRLAS (1:500; provided by Prof. Min Goo Lee at Yonsei University College of Medicine, Seoul, Korea), and a mouse anti-Kcnj10 antibody (1:500; Abnova, Taipei, Taiwan) diluted in the blocking solution, and the secondary antibody was an Alexa Fluor 555-conjugated goat anti-rabbit IgG antibody (1:1000; Invitrogen, La Jolla, CA, USA) diluted in the blocking solution. The slides were mounted with an aqueous medium (Fluoromount, Sigma-Aldrich, St. Louis, MO, USA). GFP fluorescence was detected directly. Images were captured by conventional microcopy (Axioscope, Axio Imager.A2, Carl Zeiss, Inc., Oberkochen, Germany) or confocal laser microscopy (LSM700 with ZEN 2012 software, Carl Zeiss, Inc., Oberkochen, Germany). DAPI and pendrin expressing epithelial cell in the endolymphatic sac were counted using a computer algorithm (ImageJ, National Institutes of Health, Bethesda, MD, USA). Counts were verified by manual counting. The density of pendrin expressing epithelial cells in the endolymphatic sac was determined as ratio of pendrin- and DAPI-positive cells.

#### Hematoxylin-eosin staining

Before processing the hematoxylin-eosin staining, the slides of OCT compound-embedded inner ear sections were washed with PBS, whereas the slides of paraffin-embedded inner ear sections were incubated for 1 h at 65°C, deparaffinized with xylene, and rehydrated with a graded ethanol series. All slides of the inner ears were coverslipped with mounting media (Leica Microsystems, Bensheim, Germany) and visualized using a Zeiss Axio microscope (Axio Imager.A2, Carl Zeiss, Inc., Oberkochen, Germany).

### Reverse-transcription polymerase chain reaction (RT-PCR)

The inner ears were divided by microdissection into three parts, cochlea, vestibule and endolymphatic sac and total RNA was isolated from each part according to the manufacturer's instructions (RNeasy® Micro or Mini Kit, Qiagen, Hilden, Germany). cDNAs were synthesized from the isolated total RNA (High-Capacity cDNA Reverse Transcription Kit, Applied Biosystems, Foster City, CA, USA) and expression of *Slc26a4* was analyzed by conventional and quantitative PCR (q-PCR) in 5 ng samples of cDNA.

We designed one forward primer in exon 6 of the *Slc26a4* and two reverse primers. The *Slc26a4* reverse-1 primer was located at the exon boundary between exons 7 and 8 and generated a 221 bp product with the *Slc26a4^+^* cDNA and no product with *Slc26a4^∆^* and *Slc26a4^tm1Dontuh^* cDNAs, both of which are lacking exon 8. The *Slc26a4* reverse-2 primer was located at exon 10 and generated a 478 bp product (395 bp of flanking DNA and 83 bp from the wild-type exon 8) with *Slc26a4^+^* cDNA, a 894 bp product (395 bp of flanking DNA and 499 bp of the *Neo^R^*cassette that replaced exon 8) with *Slc26a4^∆^*cDNA, and a 395 bp product (without 83 bp from the wild-type exon 8) with* Slc26a4^tm1Dontuh^* cDNA. The presence of viral vector rAAV2/1*-Slc26a4* was demonstrated by amplifying a 219 bp product in the *tGFP* gene*.* The *glyceraldehyde 3-phosphate dehydrogenase* (*Gapdh*) gene was amplified in all samples (202 bp) as a positive control. q-PCR was performed using SYBR green reagents (Applied Biosystems, Foster City, CA, USA) and a thermocycler (Step One RT-PCR, Applied Biosystems, Foster City, CA, USA). Relative *Slc26a4^+^* mRNA expression was calculated by normalizing the level of the* Slc26a4^+^* transcripts to the level of the *Gapdh* transcript in every sample and then to the normalized *Slc26a4^+^* transcript level in *Slc26a4*^+/+^ mice at age E14.5 (∆∆C_t_). ∆∆C_t_ values were used for statistical analysis. Relative fold changes were calculated (2^-(∆∆Ct)^) and graphed. Averages of three technical replicates of three biological replicates were analyzed. The following PCR primer sequences were used: *Slc26a4* forward, 5' ATC GTG CTC AAT GTT TCA ACC 3'; *Slc26a4* reverse-1, 5' GTA GCA ATT ATT GTC ACA ATC AC 3'; *Slc26a4* reverse-2, 5' ATC CAG AGA AGA CGT TGC TTA TCC 3'; *tGFP* forward, 5' CTT CTA CCA CTT CGG CAC CTA C 3'; *tGFP* reverse, 5' ATC TTG TCG GTG AAG ATC ACG 3'; *Gapdh* forward, 5' GGT GCT GAG TAT GTC GTG GA 3'; and *Gapdh* reverse, 5' CTA AGC AGT TGG TGG TGC AG 3'.

### Paint fill

The paint filling of the inner ears was performed using a method similar to that in a previous report 25. Briefly, P2 mice were anesthetized with using hypothermia on ice and sacrificed by decapitation. Heads were bisected and fixed overnight in Bodian's fixative (75% ethanol, 5% glacial acetic acid, 5% formalin, and 15% water). The bisected heads were then dehydrated in ethanol and cleared overnight in methyl salicylate. The inner ears were partially injected with white paint in methyl salicylate to visualize the cochlear duct, saccule, endolymphatic duct and sac. At least five inner ears were injected for each group presented.

### Auditory brainstem responses (ABR)

Hearing thresholds were determined in a sound-proofed room based on ABR recordings (ABR workstation - System 3, Tucker Davis Technology, Alachua, FL, USA), as previously described 26. Mice were anesthetized with a mixture of alfaxan (4 mg/100 g) and xylazine hydrochloride (0.13 mg/100 g) by i.m. injection and were placed on a heating pad. Subcutaneous needle electrodes were inserted into the vertex (channel), ipsilateral ear (reference), and contralateral ear (ground). Acoustic stimuli were applied monaurally through a speaker, and recordings were made in response to click and tone burst stimuli at frequencies of 8, 16, and 32 kHz. Stimuli were delivered at an amplitude of 90 dB sound pressure level (SPL) and amplitudes were reduced in 5 dB to determine acoustic thresholds. When no wave form was found, suggesting deaf, the threshold was set to the number 100 to allow numerical statistics.

### Endocochlear potential and endolymphatic pH

Mice were anesthetized with 4% tribromoethanol (0.014 ml/g i.p.) and sacrificed by decapitation at the end of the procedure. The round window of the cochlea was exposed via a ventral approach. Endocochlear potential (EP) and endolymphatic pH were measured by advancing a double-barreled microelectrode through the round window membrane and the basilar membrane into scala media of the cochlea using previously described protocols 14. After the microelectrode was positioned in the perilymphatic space, the surgical field was covered with liquid Sylgard 184 (Dow Corning Corporation, Midland, MI, USA) to limit dehydration and loss of tissue CO_2_. After advancing the electrode into endolymph and measurements of the transepithelial potentials and fluid pH values, the electrode was withdrawn and immediately calibrated in the surgical field 14. Standard solutions for calibration were pH 6 (130 mM NaCl, 20 mM MES), pH 7 (130 mM NaCl, 20 mM HEPES), and pH 8 (130 mM NaCl, 20 mM tricine). Data were recorded digital (Digidata 1440A and AxoScope 10, Molecular Devices, Sunnyvale, CA, USA) and analyzed using Origin 8 software (The Origin Company, Northhampton, MA, USA).

Double-barreled microelectrodes were fabricated from filament-containing glass tubing (World Precision Instruments 1B100F-4, Sarasota, FL, USA) using micropipette puller (Narishige PE-22, Tokyo, Japan). Microelectrodes were baked at 200°C for 2 h. After baking, ion barrels of the double-barreled microelectrodes were mounted on the holes in the lid of beaker, which heated to 210°C. Then, 20 µl dimethyldichlorsilane (Sigma-Aldrich, St Louis, MO, USA) was dripped into the beaker and the microelectrodes were silanized by exposing them to vaporized dimethyldichlorsilane for 30-35 seconds (s). After silanization, microelectrodes were baked at 200°C for 3 h. The tips of microelectrodes were broken to outer diameter of ~5 µm. Reference barrel of microelectrode was filled with 1M KCl and the ion selective barrel was filled at the tip with liquid ion exchanger (Hydrogen ionophore I - cocktail A, 95291, Sigma-Aldrich, St Louis, MO, USA) and back-filled with buffer solution (500 mM KCl, 20 mM HEPES, pH 7.34). Each barrel was connected to a dual channel differential electrometer (HiZ-223, Warner Instruments, Hamden, CT, USA) via Ag-AgCl wires.

### Scanning electron microscopy (SEM)

Mice were anesthetized with a mixture of alfaxan (4 mg/100 g) and xylazine hydrochloride (0.13 mg/100 g) by i.m. injection and sacrificed by decapitation. The inner ears were immediately harvested from the sacrificed mice and perfused cautiously through the oval window with a solution of 2% PFA dissolved in 0.1 M sodium cacodylate buffer (pH 7.4) containing 2.5% glutaraldehyde. The prepared specimens were immersed in the same fixative for 1 h at RT. The organ of Corti was prepared by the osmium tetroxide - thiocarbohydrazide (OTOTO) method, as described previously 24. The specimens were examined under a cold-field emission SEM (SU8220, Hitachi, Tokyo, Japan) that was operated at 15 kV.

### Rotarod

The ability of mice to balance on a revolving rod was quantified by measuring the latency to fall (47650 Rota-Rod NG, Ugo Basile, Varese, Italy). On the day of testing, the cohort in their home cages was placed in the testing room at least 30 minutes before the actual test to allow them to acclimate to the environment. For each test, mice were placed in individual lanes on the Rotarod. The initial rotation speed was set to 5 revolutions per minute (rpm) and speed was accelerated to 40 rpm over 60 s. Mice were tested on 9 consecutive days in 4 trials per day spaced by 15-minute intertrial pauses. Daily medians were calculated for each mouse. Averages and standard deviations were obtained for each group of mice. The *n* is the number of mice.

### Statistical analyses

Data were plotted directly, presented by averages ± SD or by box-plots indicating median, 25% and 75% of the data. Whiskers in box plots represent 5% and 95% of the data. Data sets that failed the Shapiro-Wilk normality test were transformed according to x_transformed_ = log (x) when data sets did not contain negative values and when data sets contained negative values according to x_transformed_ = log (x + 1 - x_min_), where x_min_ is the minimum value in the dataset. Significance of differences among groups represented by raw or transformed data sets that passed the Shapiro-Wilk normality test were determined by one-way or two-way ANOVA followed by all-pairwise multiple comparison procedures (Bonferroni t-test). Significance of differences among groups represented by data sets that did not pass the Shapiro-Wilk normality test was determined by Kruskal-Wallis one-way ANOVA on ranks followed by multiple comparisons versus a control group (Dunn's Method). Pearson correlation was used to evaluated interactions. Pearson correlations were interpreted based on ranges of the absolute R value: strong, 1.0 ≤ R ≤ 0.5; moderate: 0.5 < R ≤ 0.3; weak: 0.3 < R ≤ 0.1. Significance of data in contingency tables was determined by Fisher's exact test. Statistical analyses were assisted by software packages (SPSS Statistics 21.0, IBM, Chicago, IL, USA or Sigmastat 4.0, Systat Software, San Jose, CA, USA). *p*<0.05 was considered significant.

## Results

### Injection of rAAV2/1-Slc26a4-tGFP at E12.5 targets epithelial cells in the developing endolymphatic sac

Recombinant adenovirus rAAV2/1 vectors carrying *Slc26a4* and *tGFP* cDNAs under the control of a CMV promoter (rAAV2/1-*Slc26a4-tGFP*) were used to transfect inner ears of *Slc26a4*-deficient mice (Figure 1G). Vectors were injected *in utero* into E12.5 otocysts of two *Slc26a4*-deficient mice, knockout (*Slc26a4^∆/∆^*) and knock-in (*Slc26a4^tm1Dontuh/tm1Dontuh^*) mice. Expression of *Slc26a4* and *tGFP* transcripts in the inner ear were examined by PCR using the *Slc26a4* forward and the *Slc26a4* reverse-2 primer as well as forward and reverse *tGFP* primers (Figure 1H). The replacing form of *Slc26a4* mRNA with the *Neo^R^* cassette instead of exon 8 and the skipping form of *Slc26a4* mRNA lacking exon 8 were detected in the *Slc26a4^∆/∆^* and *Slc26a4^tm1Dontuh/tm1Dontuh^* mice, respectively (Figure 1H, upper panel, lanes 3 and 5). When we delivered rAAV2/1-*Slc26a4*-*tGFP*, the mRNA expression of wild-type *Slc26a4* mRNA was observed in the inner ear at E13.5, 24 hours after virus injection (Figure 1H, upper panel lane 4 and 6). We also determined that mRNA expression of the *tGFP* was only detectable in the inner ear of injected mice (Figure 1H, middle panel). Amplification of a 202 bp transcript representing the expression of *Gapdh* mRNA served as a control. The data demonstrate that local gene delivery induced onset of expression of *Slc26a4* and *tGFP* mRNAs by one day after injection.

**Figure 2:**
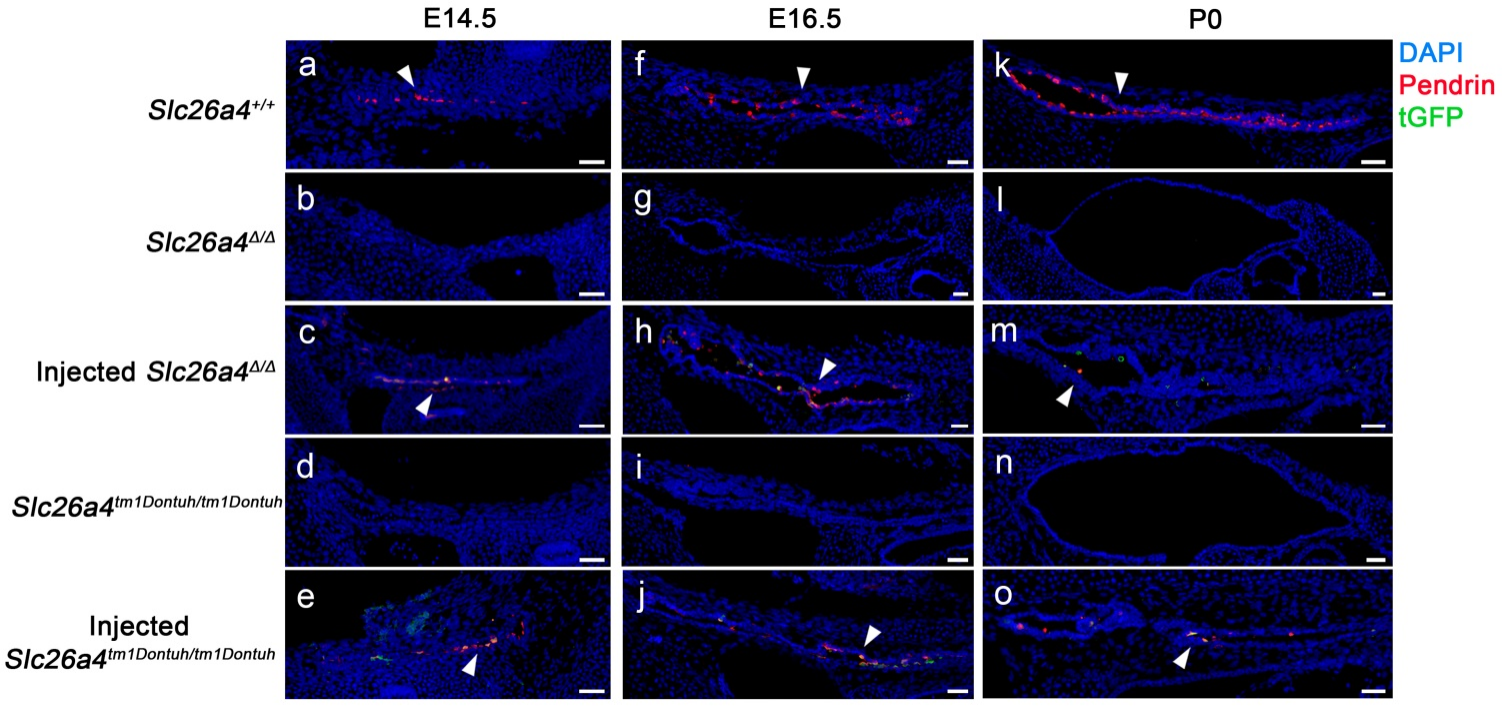
** Pendrin and tGFP protein expression in the endolymphatic sac.** Pendrin immunoreactivity (red) and direct fluorescence of tGFP (green) were evaluated in endolymphatic sacs of Slc26a4^+/+^, Slc26a4^Δ/Δ^, injected Slc26a4^Δ/Δ^, Slc26a4^tm1Dontuh/tm1Dontuh^, and injected Slc26a4^tm1Dontuh/tm1Dontuh^ mice at E14.5 (**a-e**), E16.5 (**f-j**) and P0 (**k-o**). Endogenous pendrin expression was observed in mitochondria-rich cells Slc26a4^+/+^ mice (**a, f, k**). A similar pattern of rAAV2/1-Slc26a4-tGFP vector-induced pendrin and tGFP expression was observed in injected Slc26a4^∆/∆^ mice (**c, h, m**) and injected Slc26a4^tm1Dontuh/tm1Dontuh^ mice (**e, j, o**). Nuclei were stained with DAPI (blue). White arrowheads point to representative pendrin-expressing cells. Representative images of 3 replicates each. Scale bars: 20 µm.

Expression of pendrin and tGFP protein was examined by confocal immunocytochemistry and direct fluorescence, respectively. Pendrin-positive cells were detected in the endolymphatic sac of *Slc26a4^+/+^* mice but not in *Slc26a4^∆/∆^* or *Slc26a4^tm1Dontuh/tm1Dontuh^* mice (Figure 2). Injection of rAAV2/1-*Slc26a4-tGFP* induced pendrin and tGFP expression in the endolymphatic sac (Figure 2). Whether pendrin-positive cells were mitochondria-rich or ribosomal-rich cells remained undetermined. In contrast to the expression of exogenous pendrin protein in the endolymphatic sac, no pendrin expression was detected in the cochlea or in vestibular transitional cells of injected mice (Figure S2 and S3). Ectopic expression of exogenous pendrin, however, was detected in vestibular hair cells at P10 (Figure S4). In addition, injection of rAAV2/1-*tGFP* showed poor tropism of rAAV2/1-*tGFP* to the cochlear and vestibular hair cells in both *Slc26a4*-deficient mice (Figure S5 and S6), which was consistent with tropism of rAAV2/1-*Slc26a4*-*tGFP*.

### Local gene delivery restores hearing

**Figure 3:**
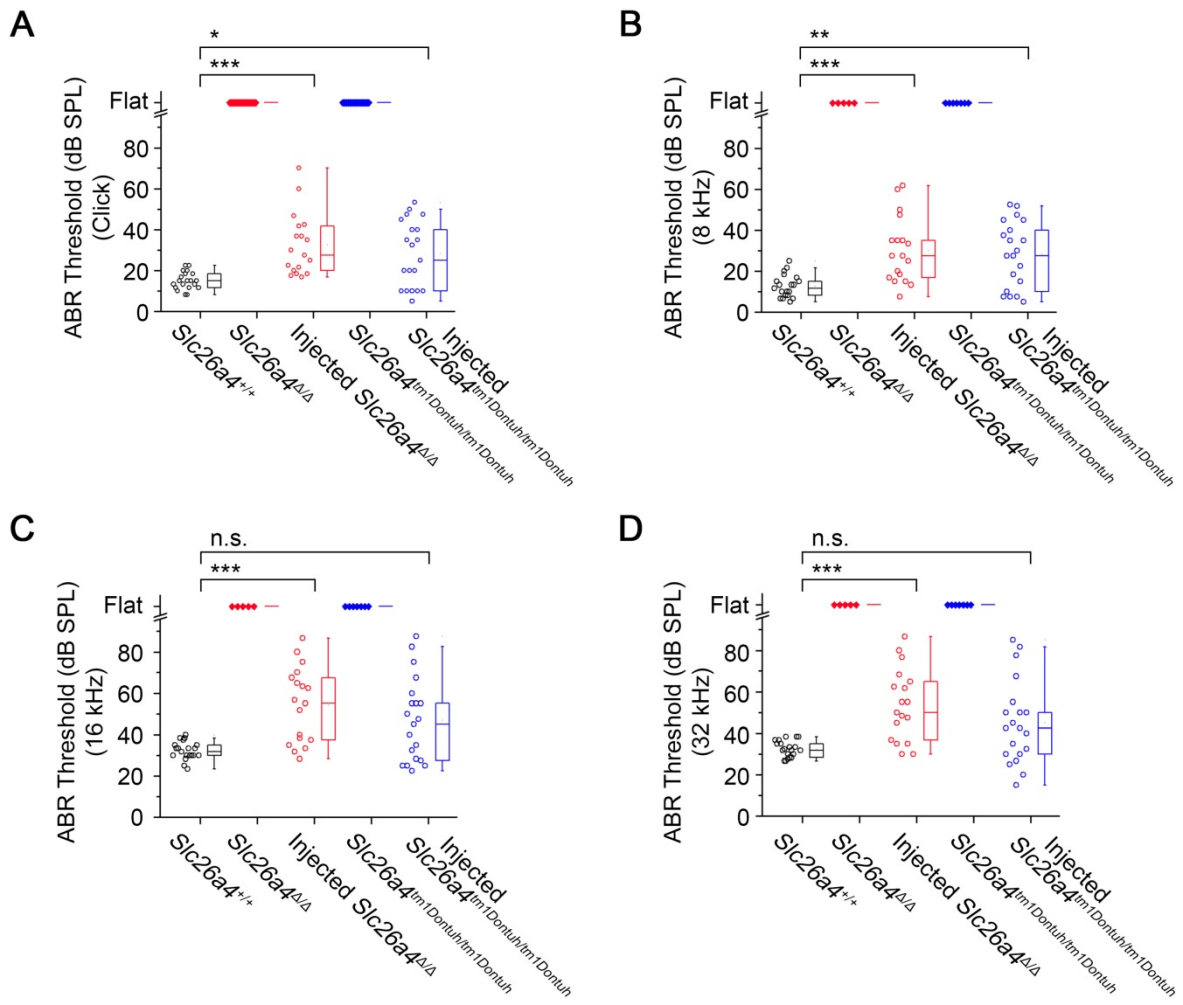
** Local gene delivery restores hearing.** Auditory brainstem response (ABR) thresholds in response to click sounds (**A**), tone burst at 8 kHz (**B**), 16 kHz (**C**) and 32 kHz (**D**) tone bursts in *Slc26a4^+/+^*, *Slc26a4^Δ/Δ^*, injected *Slc26a4^Δ/Δ^*, *Slc26a4^tm1Dontuh/tm1Dontuh^*, and injected* Slc26a4^tm1Dontuh/tm1Dontuh^* mice at 3 to 5 weeks of age. Data are drawn as symbols and represented by box-plots (25%, 50%, and 75%) with whiskers (5% and 95%). Brackets mark comparisons among types of mice. Significance was evaluated by Kruskal-Wallis one-way ANOVA on ranks with Dunn's method: n.s. no significant difference, * *p*<0.05, ** *p*<0.01, *** *p*<0.001

Hearing was evaluated at 3-5 weeks of age based on ABR thresholds in response to click and sound stimuli. *Slc26a4*-deficient *Slc26a4^∆/∆^* and *Slc26a4^tm1Dontuh/tm1Dontuh^* mice were profoundly deaf. ABR recordings appeared “flat” since characteristic waveforms were not observed, not even at the highest stimulus amplitude (90 dB). Injection of rAAV2/1-*Slc26a4-tGFP* restored ABR waveforms. Ranges of ABR thresholds obtained in injected mice overlapped with ranges in *Slc26a4^+/+^* mice, however, variances of ABR thresholds were larger in injected mice (Figure 3A). Similar observations were made in response to tone bursts at 8, 16 and 32 kHz (Figure 3B-D). Medians of ABR thresholds were 10-20 dB higher in injected compared to *Slc26a4^+/+^* mice, which is consistent with a mild hearing impairment.

To determine whether hearing restoration was induced by the injection or transfection procedure rather than the delivery of *Slc26a4*, we injected a control vector, rAAV2/1-*tGFP*, which lacks *Slc26a4*. Injection of rAAV2/1-*tGFP* failed to restore hearing in *Slc26a4*-deficient mice. Thresholds were 100 ± 0 dB SPL (*n*=3) in rAAV2/1-*tGFP* vector injected *Slc26a4^∆/∆^* mice and 100 ± 0 dB SPL (*n*=3) in rAAV2/1-*tGFP* vector injected *Slc26a4^tm1Dontuh/tm1Dontuh^* mice. These results strengthen the conclusion that local gene delivery of *Slc26a4* restores the acquisition of hearing in *Slc26a4*-deficient mice.

### Local gene delivery prevents enlargement of the membranous labyrinth and loss of hair cells

**Figure 4:**
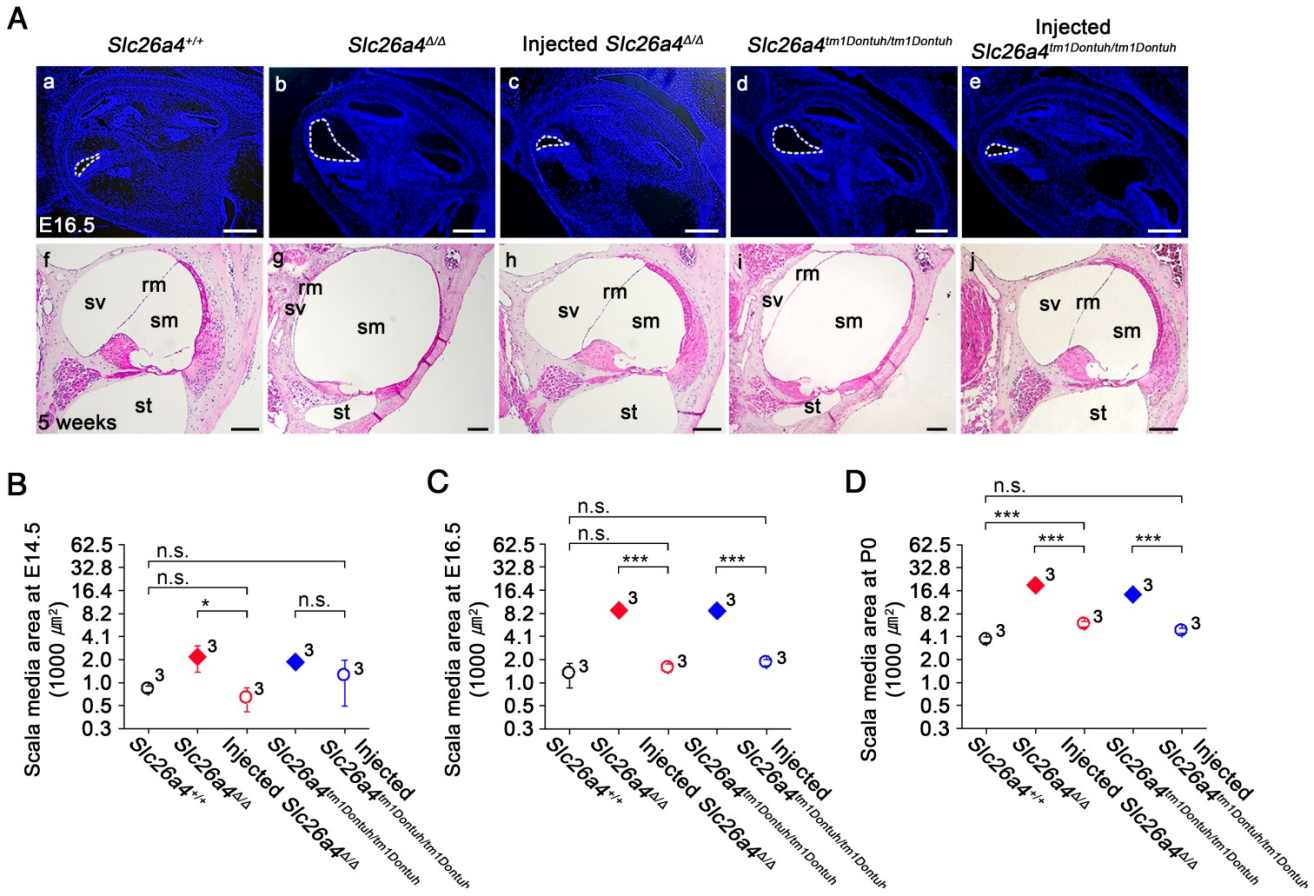
** Local gene delivery prevents enlargement of the cochlea.** (**A**) Midmodiolar sections of the cochlea from *Slc26a4^+/+^*, *Slc26a4^Δ/Δ^*, injected *Slc26a4^Δ/Δ^*, *Slc26a4^tm1Dontuh/tm1Dontuh^*, and injected* Slc26a4^tm1Dontuh/tm1Dontuh^* mice were obtained at E16.5 (**Aa-e**) and stained with DAPI (*blue*) and obtained at 5 weeks of age (**Af-j**) and stained with hematoxylin-eosin. sm, scala media; sv, scala vestibuli, st, scala tympani, rm, Reissner's membrane. Scale bars: 200 µm in (**Aa-e**) and 100 µm in (**Af-j**). (**B-D**) Quantification of cross sectional areas of scala media at E14.5 (**B**), E16.5 (**C**) and P0 (**D**). Numbers next to symbols represent the number of inner ears of the mice. Scala media was enlarged in *Slc26a4*-deficient *Slc26a4^∆/∆^* and *Slc26a4^tm1Dontuh/tm1Dontuh^* mice. Injection of rAAV2/1*-Slc26a4-tGFP* reduced the enlargement. The ears of pendrin-deficient mice shown in this figure were the contralateral ears of injected pendrin-deficient mice. Significance was evaluated by two-way ANOVA with Bonferroni t-test: n.s. no significant difference, * *p*<0.05, *** *p*<0.001.

Gross morphology of the cochlea was evaluated in histological sections and by the paint-fill method (Figure 4 and S7). Cross sectional areas of scala media of the cochlea were enlarged by a factor of 6.7 and 6.6 in E16.5 *Slc26a4^∆/∆^* and *Slc26a4^tm1Dontuh/tm1Dontuh^* mice compared to *Slc26a4^+/+^* mice, respectively (Figure 4C, see Supplementary Excel File). Injection of rAAV2/1-*Slc26a4-tGFP* reduced this enlargement and cross sectional areas differed only by factor of 0.8 and 1.5 from *Slc26a4^+/+^* mice, respectively. Similar observations were made at E14.5 and P0 (Figure 4B and D). In addition, overt enlargements of the neonatal and adult endolymphatic sac and the adult vestibular labyrinth were observed in *Slc26a4*-deficient *Slc26a4^∆/∆^* and *Slc26a4^tm1Dontuh/tm1Dontuh^* mice (Figure S7). Injection of rAAV2/1-*Slc26a4-tGFP* prevented enlargement not only of the neonatal but also the adult membranous labyrinth.

**Figure 5:**
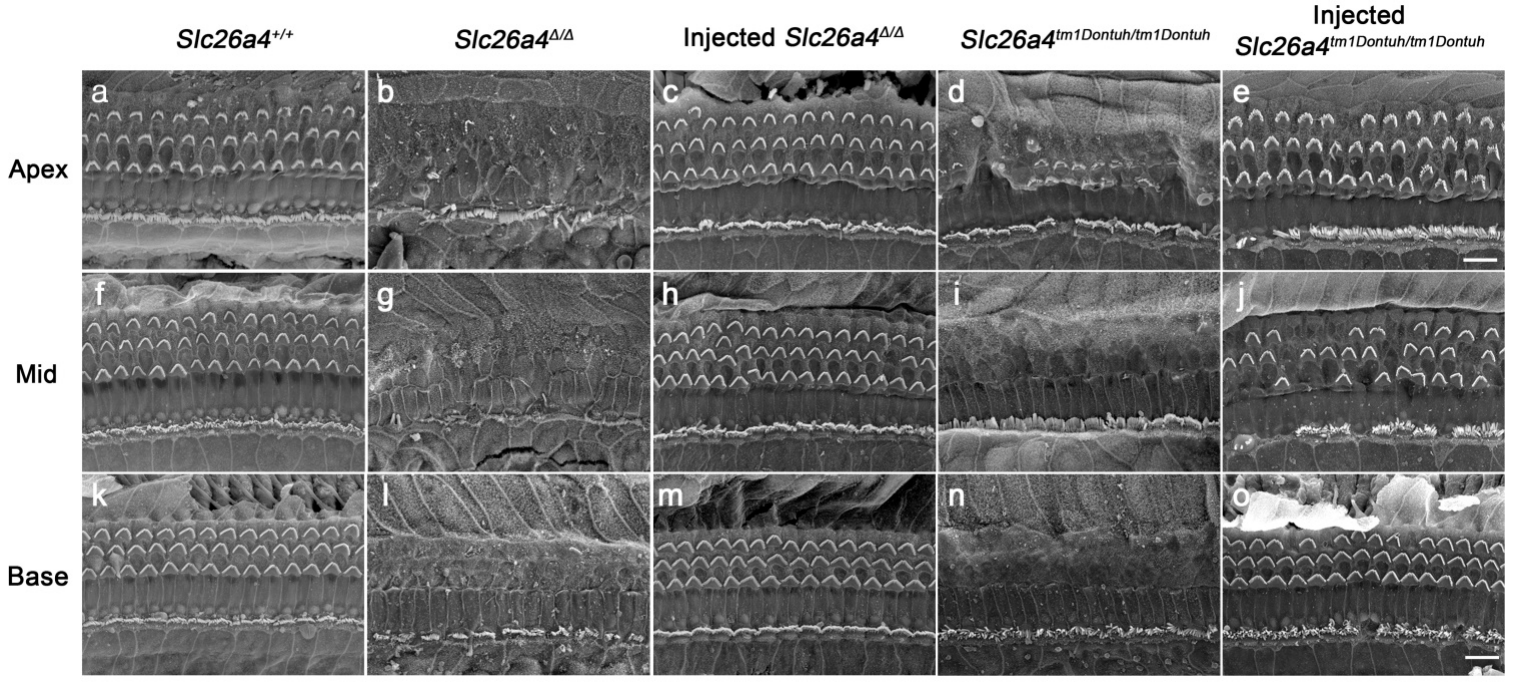
** Local gene delivery rescued outer hairs cells.** The morphology of the organ of Corti at the apex (**a-e**), mid (**f-j**) and base (**k-o**) of the cochlea was examined by scanning electron microscopy in *Slc26a4^+/+^*(**a, f, k**), *Slc26a4^Δ/Δ^* (**b, g, l**), injected *Slc26a4^Δ/Δ^* (**c, h, m**), *Slc26a4^tm1Dontuh/tm1Dontuh^*(**d, i, n**), and injected* Slc26a4^tm1Dontuh/tm1Dontuh^* mice (**e, j, o**) at 5 weeks of age. The ears of pendrin-deficient mice shown in this figure were the contralateral ears of injected pendrin-deficient mice. Representative images of 3 replicates each. Scale bar: 10 µm.

The morphological integrity of the organ of Corti at the adult stage (5 weeks) was evaluated by SEM. Severe degeneration of outer hair cells was observed in *Slc26a4*-deficient *Slc26a4^∆/∆^* and *Slc26a4^tm1Dontuh/tm1Dontuh^* mice in the basal turn of the cochlea (Figure 5). Injection of rAAV2/1-*Slc26a4-tGFP* led to the preservation of outer hair cells. Similar observations were made in the apical and middle turn of the cochlea (Figure 5).

### Local gene delivery induces Slc26a4 mRNA and transient pendrin protein expression in the endolymphatic sac

**Figure 6:**
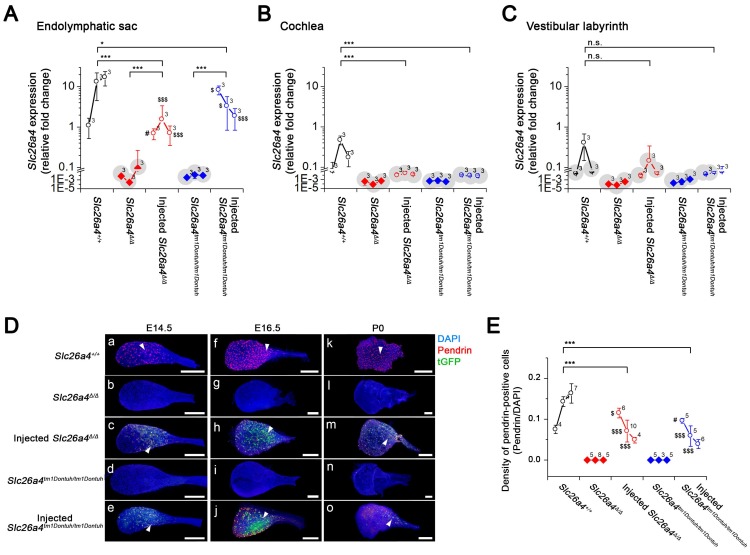
** Expression of the *Slc26a4* mRNA, Pendrin and tGFP protein in the developing inner ear.** (**A-C**) Relative *Slc26a4*^+^ expression in the endolymphatic sac, cochlea and vestibular labyrinth (mean ± SD). Numbers next to symbols represent the number of biological replicates. Transcripts of endogenous or vector-induced *Slc26a4*^+^ mRNA were quantified by qPCR. Reactions were performed on total RNA isolated from endolymphatic sacs (**A**), cochleae (**B**) and vestibular labyrinths (**C**) obtained from *Slc26a4*^+/+^, *Slc26a4*^Δ/Δ^, rAAV2/1-*Slc26a4-tGFP* injected *Slc26a4*^Δ/Δ^, *Slc26a4*^tm1Dontuh/tm1Dontuh^, and rAAV2/1-*Slc26a4-tGFP* injected *Slc26a4*^tm1Dontuh/tm1Dontuh^ mice at ages E14.5, E16.5 and P0. Relative fold changes (2^-(∆∆Ct)^) were obtained from relative *Slc26a4*^+^ mRNA expression levels (∆∆C_t_) that were calculated by normalizing the level of *Slc26a4*^+^ transcripts to the level of *Gapdh* transcripts in every sample and then normalizing *Slc26a4*^+^ transcript levels to the average level in E14.5 endolymphatic sacs of *Slc26a4*^+/+^ mice. *Slc26a4* expression levels based on C_t_ values >30 were considered undetected and marked (*grey shadow*). Significance was evaluated by two-way ANOVA with Bonferroni t-test (**A**) and by one-way ANOVA on ranks with Tukey pairwise comparison (**B-C**). Brackets mark comparisons among types of mice: n.s., no significant difference, * *p*<0.05, ** *p*<0.01, *** *p*<0.001. Expression levels in the endolymphatic sac of rAAV2/1-*Slc26a4-tGFP* injected *Slc26a4*^Δ/Δ^ and rAAV2/1-*Slc26a4-tGFP* injected *Slc26a4*^tm1Dontuh/tm1Dontuh^ mice were compared to *Slc26a4*^+/+^ mice: #, no significant difference, $ *p*<0.05, $$$ *p*<0.001. Prior to statistical analysis, the data set shown in **A** was transformed to achieve normality. The data set is available in Supplemental Excel File. (**D**) Pendrin immunoreactivity (*red*) and direct fluorescence of tGFP (*green*) were evaluated in endolymphatic sacs of *Slc26a4^+/+^*, *Slc26a4^Δ/Δ^*, injected *Slc26a4^Δ/Δ^*, *Slc26a4^tm1Dontuh/tm1Dontuh^*, and injected* Slc26a4^tm1Dontuh/tm1Dontuh^* mice at E14.5 (**Da-e**), E16.5 (**Df-j**) and P0 (**Dk-o**). Endogenous pendrin expression was observed in mitochondria-rich cells *Slc26a4^+/+^* mice (**Da,f,k**). A similar pattern of rAAV2/1-*Slc26a4*-*tGFP* vector-induced pendrin and tGFP expression was observed in injected *Slc26a4^∆/∆^* mice (**Dc,h,m**) and injected *Slc26a4^tm1Dontuh/tm1Dontuh^* mice (**De,j,o**). The ears of pendrin-deficient mice shown in this figure were the contralateral ears of injected pendrin-deficient mice. Nuclei were stained with DAPI (*blue*). White arrowheads point to representative pendrin-expressing cells. Scale bars: 200 µm. (**E**) The density of pendrin-expressing cells, obtained as ratio of pendrin-immunoreactive and DAPI-stained epithelial cells, in endolymphatic sacs obtained from *Slc26a4*^+/+^, *Slc26a4*^Δ/Δ^, rAAV2/1-*Slc26a4-tGFP* injected *Slc26a4*^Δ/Δ^, *Slc26a4*^tm1Dontuh/tm1Dontuh^, and rAAV2/1-*Slc26a4-tGFP* injected *Slc26a4*^tm1Dontuh/tm1Dontuh^ mice at ages E14.5, E16.5 and P0. Significance was evaluated by two-way ANOVA with Bonferroni t-test. Brackets mark comparisons among types of mice: *** *p*<0.001. Expression levels in the endolymphatic sac of rAAV2/1-*Slc26a4-tGFP* injected *Slc26a4*^Δ/Δ^ and rAAV2/1-*Slc26a4-tGFP* injected *Slc26a4*^tm1Dontuh/tm1Dontuh^ mice were compared to *Slc26a4*^+/+^ mice: #, no significant difference, $ *p*<0.05, $$$ *p*<0.001. The data set is available in Supplemental Excel File.

To quantify the level of *Slc26a4* mRNA and pendrin protein that was induced following delivery of the viral vector, we next performed quantitative analysis of the injected mice. Relative expression levels of *Slc26a4*^+^ mRNA were determined by qPCR using the *Slc26a4*-forward primer and *Slc26a4* reverse-1 primer, which amplified a transcript (221 bp) from endogenous or vector-induced *Slc26a4*^+^ mRNA expression (Figure 6A-C, see Supplemental Excel File). The data demonstrate that local gene delivery induced *Slc26a4*^+^ mRNA expression in the endolymphatic sac, cochlea and vestibular labyrinth.

At E14.5, densities of pendrin expressing cells in injected mice were similar or higher than in *Slc26a4^+/+^* mice (Figure 6D and E). Densities declined in injected mice from E14.5 to P0 (Figure 6E) and no pendrin expressing cells were found at the adult stage (5 weeks of age, *data not shown*).

### Local gene delivery partially rescued stria vascularis and the endocochlear potential but not the endolymphatic pH

**Figure 7:**
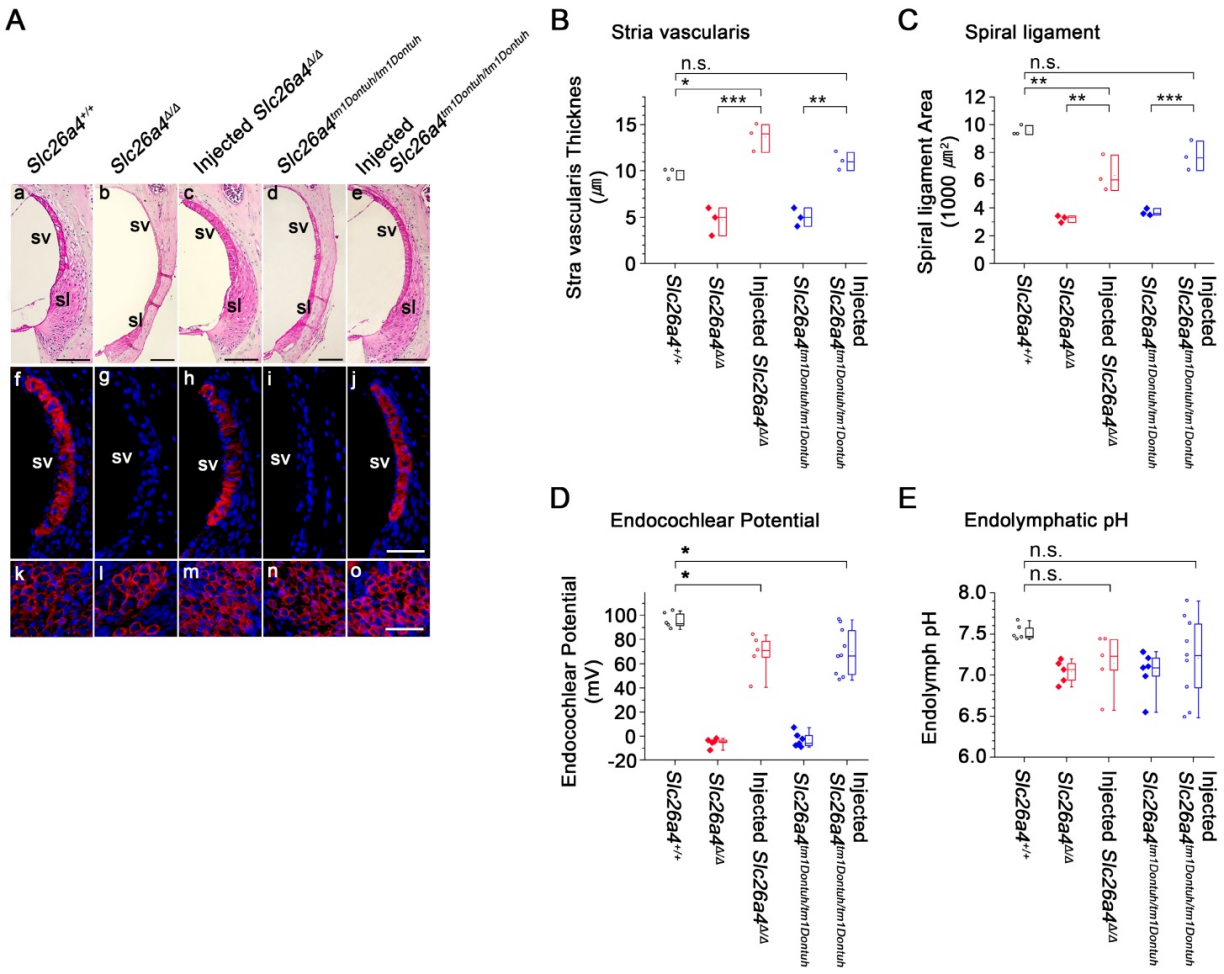
** Local gene delivery partially rescued stria vascularis and the endocochlear potential but not the endolymphatic pH.** (**A**) Sections of the lateral wall and spiral ganglion region of the cochlea were obtained from inner ears of *Slc26a4^+/+^*, *Slc26a4^Δ/Δ^*, injected *Slc26a4^Δ/Δ^*, *Slc26a4^tm1Dontuh/tm1Dontuh^*, and injected* Slc26a4^tm1Dontuh/tm1Dontuh^* mice at 5 weeks of age. (**Aa-e**) Gross morphology was evaluated in sections of the lateral wall stained with hematoxylin-eosin. (**Af-j**) KCNJ10 immunoreactivity (*red*) was evaluated in sections of the lateral wall stained with DAPI (*blue*). (**Ak-o**) Sections of the spiral ganglion region stained with DAPI (*blue*) served as a positive control for KCNJ10 immunoreactivity (*red*). SV, stria vascularis; sl, spiral ligament. Scale bars: 100 µm (**Aa-e**) and 50 µm (**Af-o**). The ears of pendrin-deficient mice shown in this figure were the contralateral ears of injected pendrin-deficient mice. (**B-C**) Thickness the stria vascularis and area of spiral ligament were measured in sections of the lateral wall. Data are drawn as symbols and represented by box-plots (25%, 50%, and 75%). Brackets mark comparisons among types of mice. Significance was evaluated by one-way ANOVA with Bonferroni t-test: n.s. no significant difference, * *p*<0.05, ** *p*<0.01, *** *p*<0.001. (**D-E**) Endocochlear potential and endolymphatic pH were measured in the inner ears of *Slc26a4^+/+^*, *Slc26a4^Δ/Δ^*, injected *Slc26a4^Δ/Δ^*, *Slc26a4^tm1Dontuh/tm1Dontuh^*, and injected* Slc26a4^tm1Dontuh/tm1Dontuh^* mice at 5 weeks of age. Data are drawn as symbols and represented by box-plots (25%, 50%, and 75%) with whiskers (5% and 95%). Brackets mark comparisons among types of mice. Significance was evaluated by two-way ANOVA with Bonferroni t-test: n.s. no significant difference, * *p*<0.05.

Stria vascularis and spiral ligament were evaluated in histologic sections and by immunocytochemistry of KCNJ10, the K^+^ channel that generates the endocochlear potential. Atrophy of stria vascularis and spiral ligament was observed in *Slc26a4*-deficient *Slc26a4^∆/∆^* and *Slc26a4^tm1Dontuh/tm1Dontuh^* mice, which resulted in a loss of KCNJ10 expression in stria vascularis (Figure 7Af-j), a reduced thickness of stria vascularis (Figure 7B), and a reduced area of spiral ligament (Figure 7C). Expression of KCNJ10 in spiral ganglion satellite cells served as a positive control (Figure 7Ak-o). Injection of rAAV2/1-*Slc26a4* largely restored the gross morphology of stria vascularis and spiral ligament in* Slc26a4*-deficient mice. No overt difference in the expression of KCNJ10 was observed between injected *Slc26a4^∆/∆^*, injected *Slc26a4^tm1Dontuh/tm1Dontuh^* and *Slc26a4^+/+^* mice. Thicknesses of stria vascularis and cross sectional areas of spiral ligament were not significantly different between injected *Slc26a4^tm1Dontuh/tm1Dontuh^* mice and *Slc26a4^+/+^* mice, however, thicknesses of stria vascularis were larger and cross sectional areas of spiral ligament were smaller in injected *Slc26a4^∆/∆^* mice compared to *Slc26a4^+/+^* mice (Figure 7B and C).

The endocochlear potential and endolymphatic pH were measured with double-barreled ion-selective microelectrodes in 5-6 week old mice (Figure 7D and E). The endocochlear potential was lost in* Slc26a4^∆/∆^* and *Slc26a4^tm1Dontuh/tm1Dontuh^* mice and endolymph was more acidic. Injection of rAAV2/1-*Slc26a4-tGFP* partially restored the endocochlear potential. Injection increased endolymphatic potentials by approximately 70 mV, however, potentials remained approximately 20 mV lower than in *Slc26a4^+/+^* mice. Endolymphatic pH values in injected* Slc26a4*-deficient mice were highly variable possibly indicating variability in the degree of restoration or instability in the pH homeostasis (Figure 7E). No significant difference was found between the endolymphatic pH of injected *Slc26a4^∆/∆^* mice or injected *Slc26a4^tm1Dontuh/tm1Dontuh^* mice and *Slc26a4^+/+^* mice, however, the power of this test was low (P=0.086), and thus the observation cannot be taken as evidence that injection of rAAV2/1-*Slc26a4-tGFP* restores the endolymphatic pH.

### The restored hearing phenotype is unstable

**Figure 8:**
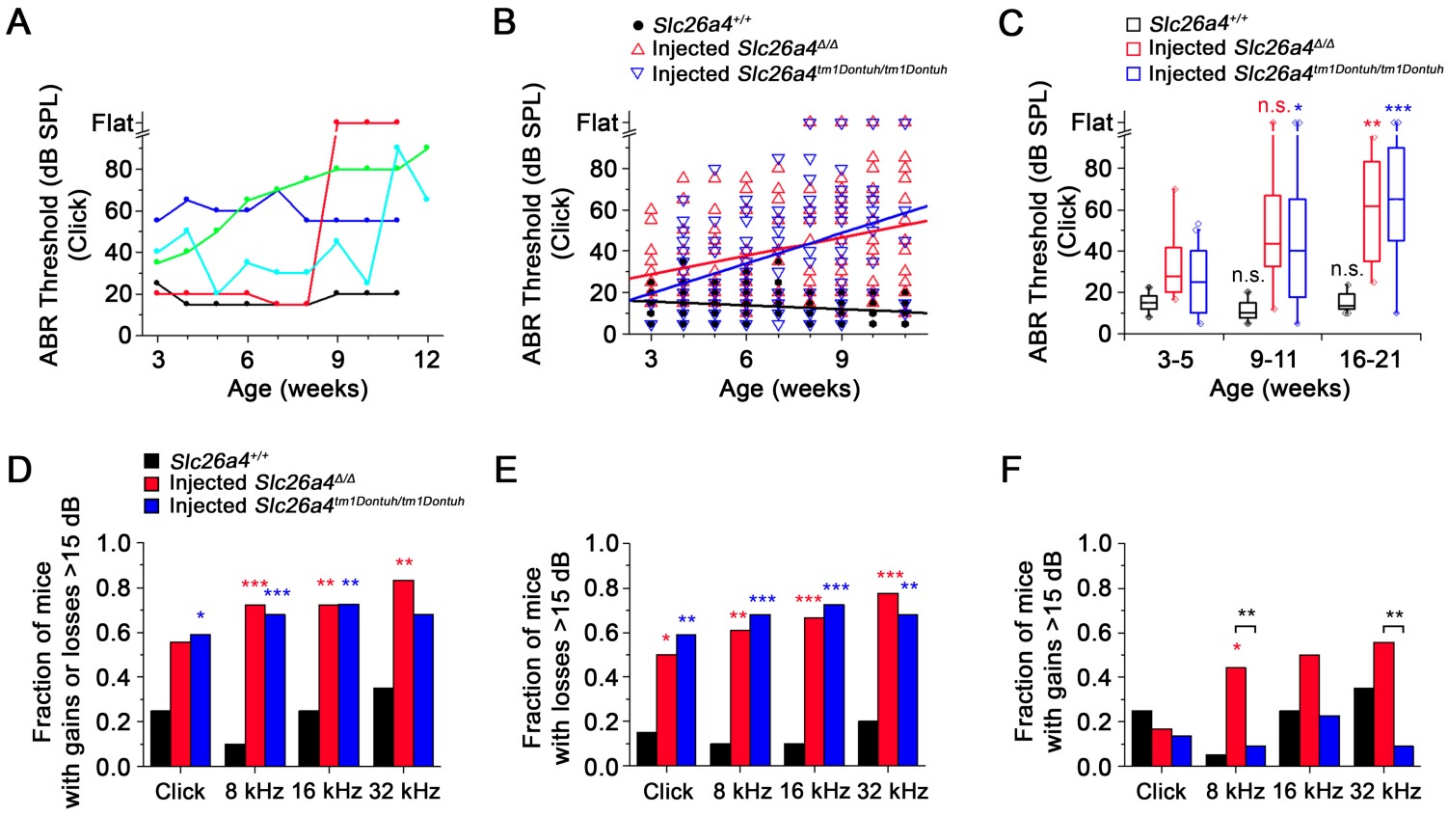
** Restored hearing phenotype is unstable.** (**A**) ABR thresholds in response to click sounds were obtained in weekly intervals. Examples from five injected *Slc26a4^∆/∆^* mice that have normal stable hearing (*black*), stable hearing loss (*blue*), progressive hearing loss (*green*), sudden hearing loss (*red*) and fluctuating hearing loss (*cyan*). (**B**) Linear regressions of ABR thresholds in response to click stimuli were obtained in *Slc26a4^+/+^* mice, injected *Slc26a4^∆/∆^* and injected *Slc26a4^tm1Dontuh/tm1Dontuh^* mice between 3 and 11 weeks of age. Slopes and Pearson's R value were -0.6 dB/week and R=0.259, 2.9 dB/week and R=0.323, and 4.9 dB/week and 0.391, respectively. (**C**) ABR thresholds in response to click stimuli were grouped into three age ranges, 3-5 weeks (*Slc26a4^+/+^* n=20, injected *Slc26a4^∆/∆^* n=18, injected *Slc26a4^tm1Dontuh/tm1Dontuh^* n=16), 9-11 weeks (*Slc26a4^+/+^* n=17, injected *Slc26a4^∆/∆^* n=17, injected *Slc26a4^tm1Dontuh/tm1Dontuh^* n=10), and 16-21 weeks (*Slc26a4^+/+^* n=10, injected *Slc26a4^∆/∆^* n=4, injected *Slc26a4^tm1Dontuh/tm1Dontuh^* n=3) to evaluate the stability of hearing in *Slc26a4^+/+^*, injected *Slc26a4^∆/∆^* mice and injected *Slc26a4^tm1Dontuh/tm1Dontuh^* mice. Data are represented by box-plots (25%, 50%, and 75%) with whiskers (5% and 95%). Outliers were drawn as symbols (*diamonds*). Differences toward the 3-5 week group were evaluated either by one-way ANOVA with Bonferroni t-test or by Kruskal-Wallis one-way ANOVA on ranks and Dunn's method, n.s. no significant difference, * *p*<0.05, ** *p*<0.01, *** *p*<0.001. (**D-F**) Fraction of mice that experienced gains or losses (**D**), losses (**E**), or gains >15 dB (**F**) were obtained and differences between injected *Slc26a4^∆/∆^* or injected *Slc26a4^tm1Dontuh/tm1Dontuh^* mice and *Slc26a4^+/+^* mice were evaluated by Fisher's exact test, * *p*<0.05, ** *p*<0.01, *** *p*<0.001. Brackets indicate significant differences between injected *Slc26a4^∆/∆^* and injected *Slc26a4^tm1Dontuh/tm1Dontuh^* mice, ** *p*<0.01.

ABR thresholds were obtained in weekly intervals to evaluate the hearing phenotype. Phenotypes of injected mice varied among individuals. Individuals with stable normal hearing as well as with stable hearing loss, sudden, progressive and fluctuating hearing loss were observed (Figure 8A). Between 3 and 11 weeks of age, ABR thresholds in response to click sound stimuli were moderately correlated with age and rose at a rate of 2.9 dB/week in injected* Slc26a4^∆/∆^* mice and at a rate of 4.9 dB/week in injected *Slc26a4^tm1Dontuh/tm1Dontuh^* mice. In contrast, ABR thresholds were only weekly correlated with age in *Slc26a4^+/+^* mice (Figure 8B). Similar observations were made in response to tone burst stimuli (Figure S8A). Data were grouped by age to determine significance. Medians of hearing thresholds in response to click stimuli in injected mice at 16-21 weeks of age were significantly higher than at 3-5 weeks, whereas thresholds in *Slc26a4^+/+^* mice were not significantly different (Figure 8C). No significant differences were found between injected *Slc26a4^∆/∆^* and injected *Slc26a4^tm1Dontuh/tm1Dontuh^* mice. Similar observations were made in response to tone burst stimuli (Figure S8B). These data demonstrate progressive hearing loss in injected *Slc26a4^∆/∆^* and injected *Slc26a4^tm1Dontuh/tm1Dontuh^* mice.

Variances of ABR thresholds were larger in injected mice than in *Slc26a4^+/+^*mice, which suggests that hearing is fluctuating. To detect hearing fluctuations, differences between successive weekly threshold measurements were computed (Figure S9). Differences larger than ±15 dB were considered fluctuations consisting of either a loss or a gain of hearing. Contingency tables of counts of animals that do or do not experienced fluctuations were evaluated using Fisher's exact test. The fraction of mice that experienced fluctuations was larger in injected mice than in *Slc26a4^+/+^* mice (Figure 8D). No significant differences were found between injected *Slc26a4^∆/∆^* and injected *Slc26a4^tm1Dontuh/tm1Dontuh^* mice. The average frequency of fluctuations was 1 per 5 weeks in injected *Slc26a4^∆/∆^* and injected *Slc26a4^tm1Dontuh/tm1Dontuh^* mice and 1 per 19 weeks in *Slc26a4^+/+^* mice. Moreover, the fraction of mice that experienced losses >15 dB was greater in injected mice than in *Slc26a4^+/+^* mice with no significant difference between the two injected mice (Figure 8E). Injected *Slc26a4^∆/∆^* mice, however, experienced more gains >15 dB than injected *Slc26a4^tm1Dontuh/tm1Dontuh^* mice, although, this observation was supported only by measurements in response to 8 and 32 kHz tone burst stimuli (Figure 8F). The average frequency of hearing gains was 1 per 16 weeks in injected *Slc26a4^∆/∆^* and 1 per 32 weeks in injected *Slc26a4^tm1Dontuh/tm1Dontuh^* mice. Taken together, the data demonstrate considerable variability in the individual hearing phenotype of injected mice and overall a fluctuating and progressive hearing loss phenotype. The lower rate of hearing loss progression and higher frequency of hearing gains in *Slc26a4^∆/∆^* mice compared to *Slc26a4^tm1Dontuh/tm1Dontuh^* mice may be an effect of the genetic background. The background strain of the *Slc26a4^∆/∆^* mice, 129S6, is less prone to age- and noise-induced hearing loss than the C57BL/6 strain, which provides the background of the *Slc26a4^tm1Dontuh/tm1Dontuh^* mice 27.

### Loss of outer hair cells in mice with profound hearing loss

Injected *Slc26a4*-deficient mice with profound hearing loss were evaluated by histology and by immunocytochemistry. No enlargement of scala media was observed in a deaf 26 week old injected *Slc26a4^∆/∆^* mouse, and in two deaf 10 week old and an 11 week old injected *Slc26a4^tm1Dontuh/tm1Dontuh^* mice (Figure S10A). Most notably, the loss of outer hair cells was observed (Figure S10B). No overt degeneration of stria vascularis or loss of KCNJ10 expression were observed (Figure S10C). These observations suggest that profound deafness in injected *Slc26a4*-deficient mice was permanent.

### Local gene delivery fails to restore vestibular function

**Figure 9:**
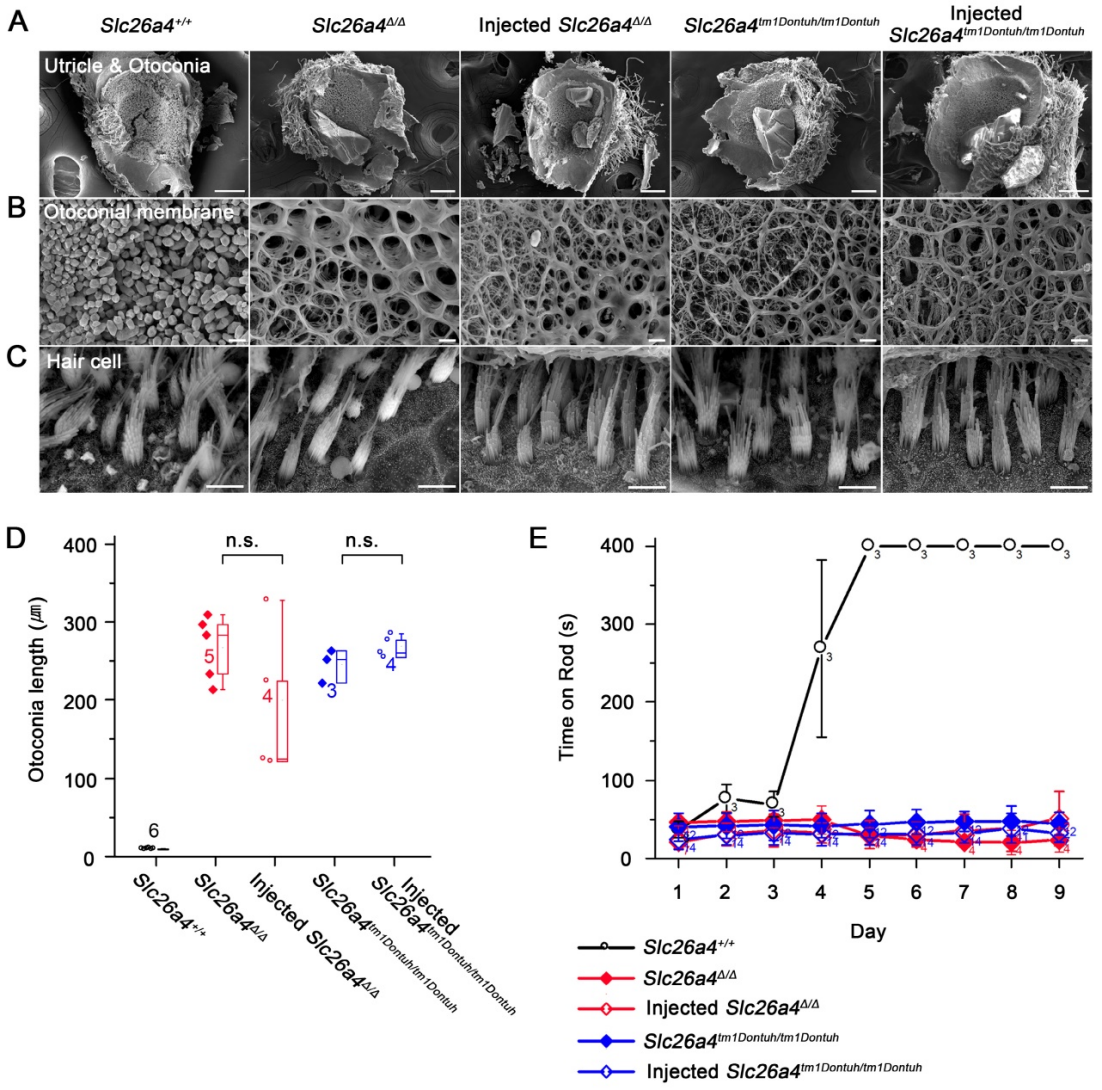
** Local gene delivery fails to restore otoconia and to rescue vestibular function.** (**A-C**) The morphology of the otoconia in the utricle (**A**), the otoconia membrane (**B**) and the vestibular hair cells (**C**) were examined by scanning electron microscopy in the *Slc26a4^+/+^*, *Slc26a4^Δ/Δ^*, injected *Slc26a4^Δ/Δ^*, *Slc26a4^tm1Dontuh/tm1Dontuh^*, and injected* Slc26a4^tm1Dontuh/tm1Dontuh^* mice at 5 weeks of age. The ears of pendrin-deficient mice shown in this figure were the contralateral ears of injected pendrin-deficient mice. Representative images of 3 replicates each. Scale bars: 100 µm in (**A**), 5 µm in (**B**) and (**C**). (**D**) Quantification of otoconia length in the inner ears of the *Slc26a4^+/+^*, *Slc26a4^Δ/Δ^*, injected *Slc26a4^Δ/Δ^*, *Slc26a4^tm1Dontuh/tm1Dontuh^*, and injected* Slc26a4^tm1Dontuh/tm1Dontuh^* mice at 5 weeks of age. Data are drawn as symbols and represented by box-plots (25%, 50%, and 75%) with whiskers (5% and 95%). Brackets mark comparisons among types of mice. Significance was evaluated by one-way ANOVA: n.s. no significant difference. (**E**) Rotarod tests, which evaluated whether a mouse can balance up to 400s on an accelerating revolving rod, were performed in *Slc26a4^+/+^*, *Slc26a4^Δ/Δ^*, injected *Slc26a4^Δ/Δ^*, *Slc26a4^tm1Dontuh/tm1Dontuh^*, and injected* Slc26a4^tm1Dontuh/tm1Dontuh^* mice at 5 weeks of age.

The gross morphology of vestibular tissues was evaluated histologically and by SEM. Otoconia were replaced with mega-otoconia in *Slc26a4^∆/∆^* and *Slc26a4^tm1Dontuh/tm1Dontuh^* mice (Figure 9A). Mega-otoconia were also observed in injected mice (Figure 9A and D). No overt differences between *Slc26a4^+/+^* mice, injected and uninjected *Slc26a4^∆/∆^* and *Slc26a4^tm1Dontuh/tm1Dontuh^* mice were observed in the structure of the otoconia membrane and in the appearance of vestibular hair cells (Figure 9B and C). Further, no overt differences were observed in the histology of the saccule, utricle, and crista ampullaris (Figure S11). Vestibular function was evaluated by the rotarod test, which measures the ability of an animal to maintain balance on a rotating rod. After a 4 to 5 days of training, 5-week-old *Slc26a4^+/+^*mice were able to balance on the accelerating rod. Neither *Slc26a4^∆/∆^* and *Slc26a4^tm1Dontuh/tm1Dontuh^* mice nor injected *Slc26a4^∆/∆^* or injected *Slc26a4^tm1Dontuh/tm1Dontuh^* mice were able to balance on the rod (Figure 9E). These observations demonstrate that local gene delivery failed to restore otoconia and vestibular function in *Slc26a4*-deficient mice.

## Discussion

The most salient findings of this study are that local AAV-mediated delivery of *Slc26a4* causes transient pendrin expression in the endolymphatic sac, which prevents enlargement of the membranous labyrinth and that *Slc26a4* delivery restores the acquisition of hearing but fails to restore a stable hearing phenotype and fails to restore otoconia formation and the acquisition of vestibular function.

### Local gene delivery induces transient pendrin expression in the endolymphatic sac

Pendrin expression in *Slc26a4^+/+^*mice begins at E11.5 in the endolymphatic sac, at E14.5 in outer sulcus epithelial cells in the cochlea and in transitional cells of the utricle and saccule, and at E16.5 in transitional cells of vestibular ampullae. At all of these sites, pendrin expression is maintained in *Slc26a4^+/+^*mice throughout adulthood 12, 16. Injection of rAAV2/1-*Slc26a4-tGFP* into E12.5 otocysts of *Slc26a4*-deficient mice induced *Slc26a4*^+^ mRNA expression and transient pendrin protein expression in the endolymphatic sac (Figure 6). The observation that injection induced no protein expression in the cochlea and no protein expression in vestibular transitional cells (Figure S2 and S3) is consistent with the tropism of the vector. A similar expression pattern was observed after injection of the rAAV2/1-*GFP* into E12.5 otocysts (Figure S5 and S6).

### Hearing and vestibular function differ in their requirements of pendrin expression

Studies in different mouse models suggested that a temporally and spatially limited expression of pendrin is sufficient to restore normal hearing and balance in *Slc26a4*-deficient mice. Li *et al* demonstrated in transgenic *Atp6v1b1-SLC26A4;Slc26a4^∆/∆^* mice that pendrin expression spatially limited to the endolymphatic sac is sufficient to restore normal hearing and vestibular function 22. In contrast, Choi *et al.* had developed double-transgenic *Slc26a4^∆/∆^* mice and proposed that the temporally limited expression of pendrin from E16.5 to P2 is sufficient for normal hearing 21. Two sets of experiments consisting of a delayed onset of pendrin expression and a premature termination of pendrin expression bracketed a seven-day period between E16.5 to P2 that appeared to be critical for the development of normal hearing. Whether this seven-day period was sufficient to restore vestibular function remained undetermined 21. The present study is the first to implement temporally and spatially limited expression simultaneously. Our protocol for gene delivery induced pendrin expression nearly exclusively in the endolymphatic sac. Expression did not fully cover the critical period between E16.5 to P2. Transient pendrin expression prevented enlargement of the membranous labyrinth and restored hearing but failed to restore otoconia formation and vestibular function (Figure 3, 4, and 9). Mega-otoconia may be the result of a lower endolymphatic pH and elevated endolymphatic Ca^2+^ concentrations 13. The data demonstrate that development of hearing and vestibular function differs in the requirement for pendrin expression. Normal development of vestibular function appears to require an earlier onset, a higher level or a longer period of pendrin expression in the endolymphatic sac.

### Pendrin expression in the endolymphatic sac promotes the acquisition of a stable hearing phenotype

Transient pendrin expression in injected *Slc26a4^∆/∆^* and injected *Slc26a4^tm1Dontuh/tm1Dontuh^* mice prevented enlargement of the membranous labyrinth and restored hearing with average thresholds at 3-5 weeks of age that were approximately 10-20 dB above thresholds in *Slc26a4^+/+^*mice consistent with a mild hearing loss (Figure 3 and 4). Similar observations had been made in double-transgenic *Slc26a4^∆/∆^* mice in which pendrin expression was discontinued at E17.5 (DE17.5-*Slc26a4^∆/∆^* mice), which is six days before the end of the critical period 28. Limited pendrin expression in injected *Slc26a4^∆/∆^* and injected *Slc26a4^tm1Dontuh/tm1Dontuh^* mice and in DE17.5-*Slc26a4^∆/∆^* mice had similar consequences including a similar degree hearing loss, similar effects on the endocochlear potential, and the stability of hearing thresholds. In both models, the endolymphatic potential was reduced and measured 60 or 70 mV rather than 100 mV as observed in* Slc26a4^+/+^*mice (Figure 7D). In DE17.5-*Slc26a4^∆/∆^* mice the pH in cochlear endolymph was lower than in *Slc26a4^+/+^* mice, and in both models variances of ABR thresholds were higher than in *Slc26a4^+/+^* due to hearing fluctuations (Figure 3) 28. Interestingly, a longer period of pendrin expression in the endolymphatic sac may be more important for the acquisition of a stable hearing phenotype than local expression of pendrin in the cochlea since hearing thresholds were normal and hearing was stable in *Atp6v1b1-SLC26A4;Slc26a4^∆/∆^* mice, which lack pendrin expression in the cochlea but sustain pendrin expression in the endolymphatic sac 22.

### Pendrin expression in spindle-shaped cells stabilizes hearing

The overall progression of hearing loss was notably faster in injected *Slc26a4^∆/∆^* mice (2.9 dB/week) and injected *Slc26a4^tm1Dontuh/tm1Dontuh^* mice (4.9 dB/week, Figure 8B) than in DE17.5-*Slc26a4^∆/∆^* mice (0.4 dB/week) 28. A key difference between these models is that injected *Slc26a4^∆/∆^* mice and injected *Slc26a4^tm1Dontuh/tm1Dontuh^* mice lacked pendrin expression in spindle-shaped cells in the lateral wall of the cochlea (Figure S2) whereas DE17.5-*Slc26a4^∆/∆^* mice expressed pendrin in spindle-shaped cells at a level of 10% of that observed in *Slc26a4^+/+^*mice 29. The concept that pendrin expression in spindle-shaped cells affects the frequency of fluctuations and the progression of hearing loss is supported by the finding that hearing thresholds in DE17.5-*Slc26a4^∆/∆^* mice were lower and fluctuations diminished, when pendrin expression in spindle-shaped cells was elevated to 30% of that observed in *Slc26a4^+/+^*mice 29. Pendrin expression in spindle-shaped cells may contribute to the homeostasis of stria vascularis and this contribution may be more important for hearing in mice with a lower endolymphatic pH such as DE17.5-*Slc26a4^∆/∆^* mice than in mice with a normal endolymphatic pH such as *Atp6v1b1-SLC26A4;Slc26a4^∆/∆^* mice 22. It is conceivable that rates of pendrin-mediated HCO_3_^-^ secretion and Cl^-^ absorption across the apical membrane of spindle-shaped cells are elevated at lower endolymphatic pH values, when the endolymphatic HCO_3_^-^ concentration is lower and the HCO_3_^-^ gradient steeper.

### Feasibility of gene therapy

Our protocol of AAV2/1-*Slc26a4-tGFP* injection induced a transient pendrin protein expression nearly exclusively in the endolymphatic sac. This limited pendrin expression was sufficient to restore the acquisition of hearing but failed to restore a stable hearing phenotype and the acquisition of vestibular function. Earlier onset, longer periods or higher levels of pendrin expression in the endolymphatic sac may be necessary to fully restore cochlear and vestibular function. Our study suggests that gene therapy for hearing loss caused by *SLC26A4* mutations in human patients is feasible, however, ethical concerns should prevent a direct translation, since local gene delivery amounts to an invasive procedure for a non-lethal disease. It will be necessary to develop non-invasive methods that induce sufficient pendrin function to ensure the development of robust cochlear and vestibular phenotypes.

## Supplementary Material

Supplementary figures and tables.

Supplementary excel for hearing function.

Supplementary excel for statistics.

## Abbreviations

ABR: auditory brainstem responses
CMV: cytomegalovirus
DAPI: 4'-6-diamidino-2-phenylindole
dB: decibel
E: embryonic day
EDTA: ethylenediaminetetraacetic acid
EVA: enlarged vestibular aqueduct
GAPDH: glyceraldehyde 3-phosphate dehydrogenase
GFP: green fluorescent protein
h: hours
I.M.: intramuscular
ITR: inverted terminal repeat
OCT: optimal cutting temperature
P: postnatal day
PBS: phosphate buffered saline
PFA: paraformaldehyde
q-PCR: quantitative polymerase chain reaction
rAAV: recombinant adeno-associated virus
RPM: revolutions per minute
RT: room temperature
RT-PCR: reverse-transcription polymerase chain reaction
s: seconds
SEM: scanning electron microscopy
SLC26A4: solute carrier family 26 member 4
SPL: sound pressure level
TCH: thiocarbohydrazide
tGFP: turbo green fluorescent protein
