# Supplementary material for: Gene therapy for hereditary hearing loss by SLC26A4 mutations in mice reveals distinct functional roles of pendrin in normal hearing
Source: Theranostics. 2019 Sep 23;9(24):7184–99. doi: 10.7150/thno.38032 (PMC6831294; doi:10.7150/thno.38032)
Supplement: Supplementary file 1 — Supplementary figures and tables. [file thnov09p7184s1.pdf]

Supplementary figures

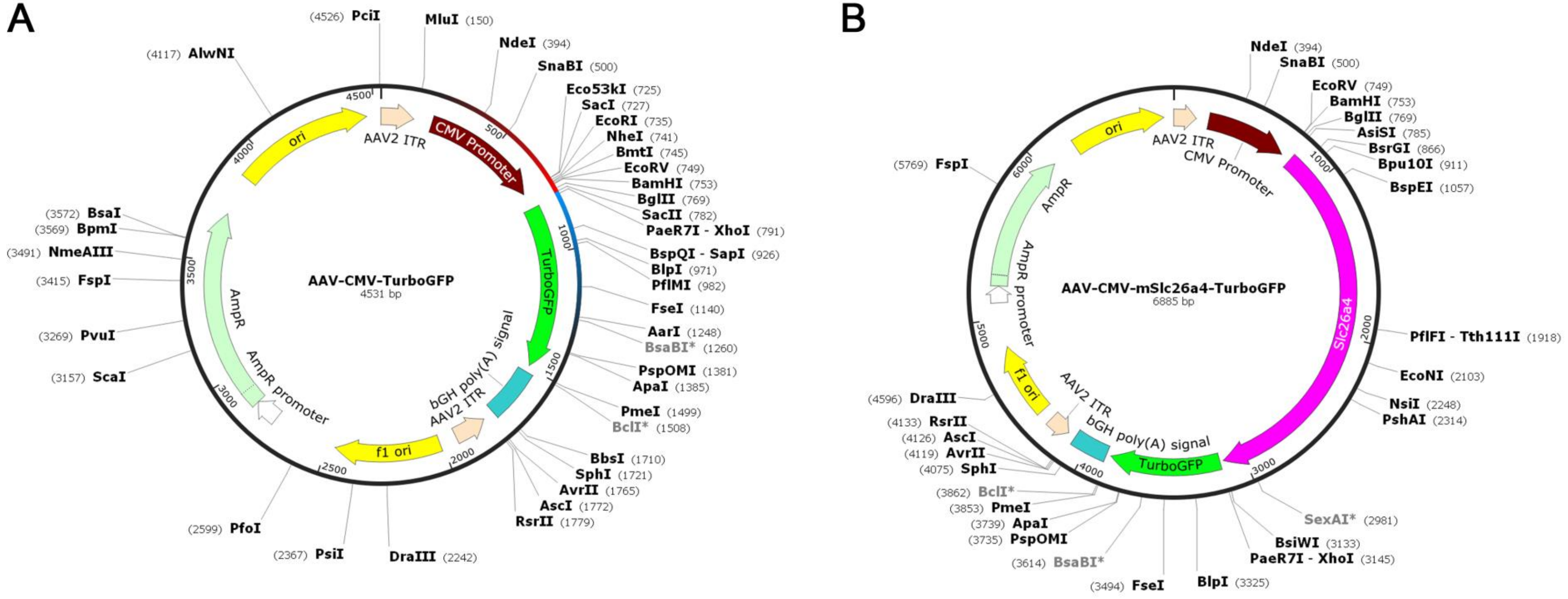

Supplementary Figure 1. **The detailed vector maps of rAAV2/1-*tGFP* and rAAV2/1-*Slc26a4-tGFP*.**

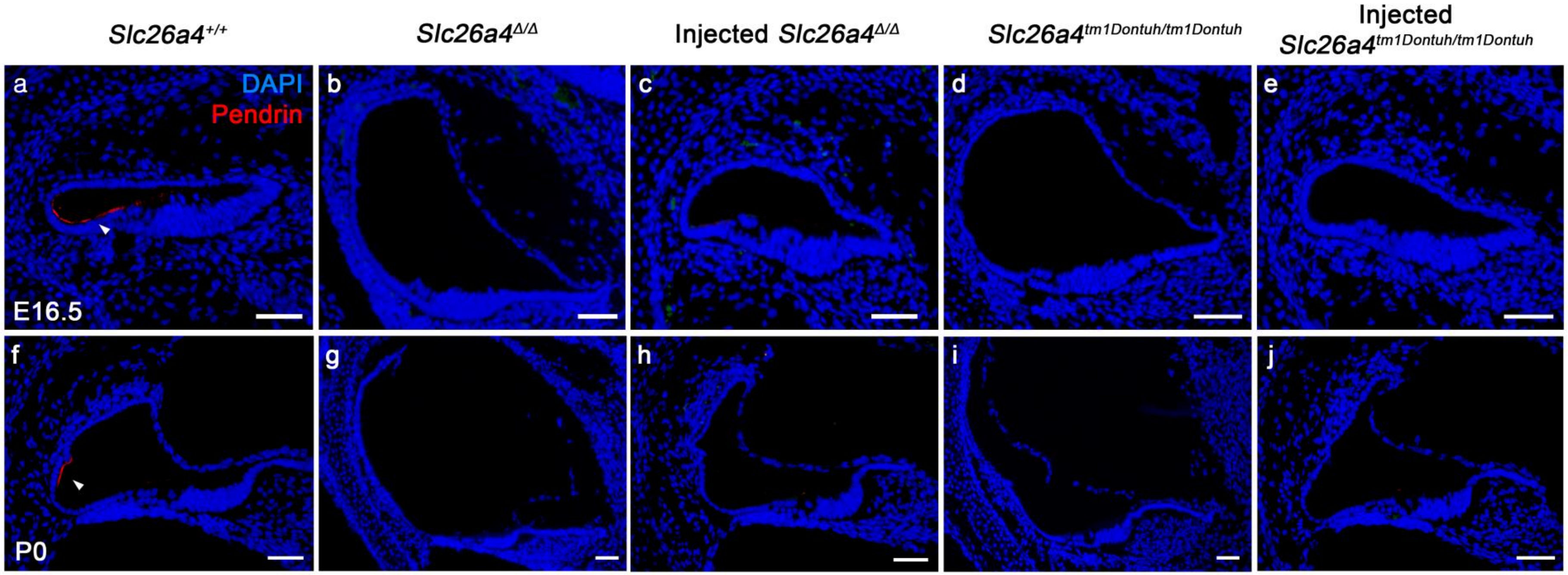

Supplementary Figure 2. **Pendrin expression in the cochlea.**

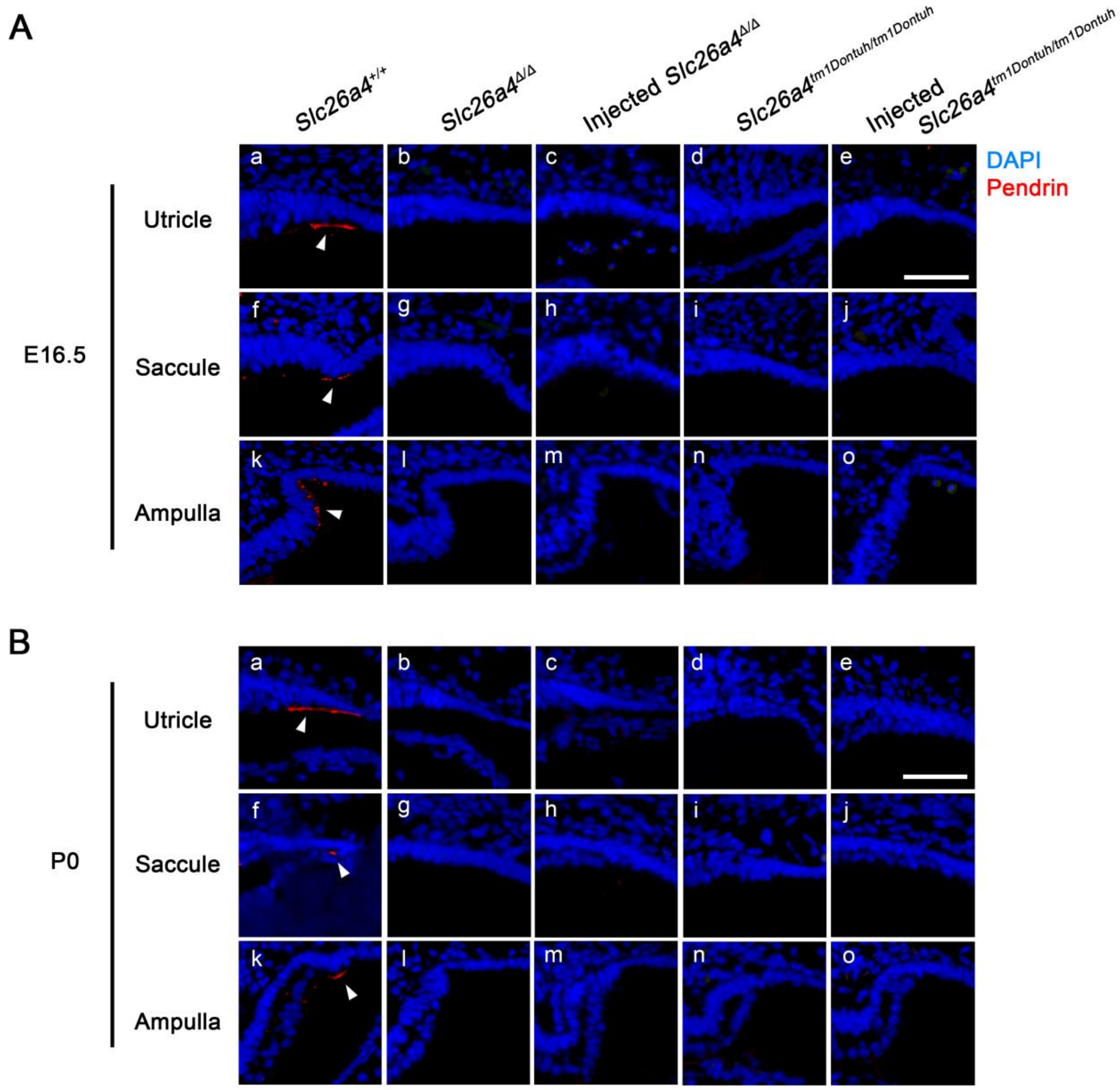

Supplementary Figure 3. **Pendrin expression in vestibular transitional cells.**

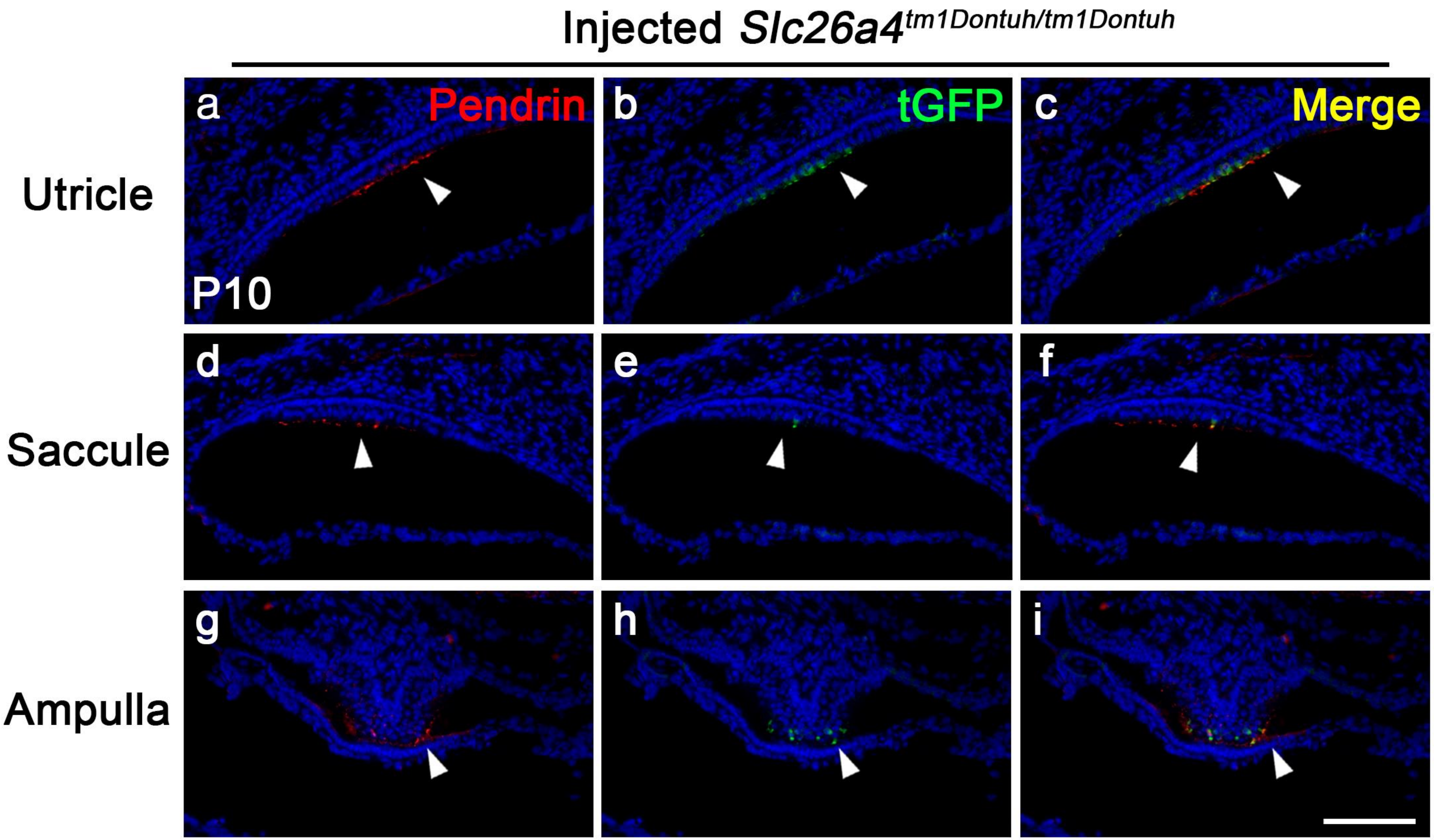

Supplementary Figure 4. **Pendrin expression in vestibular hair cells.**

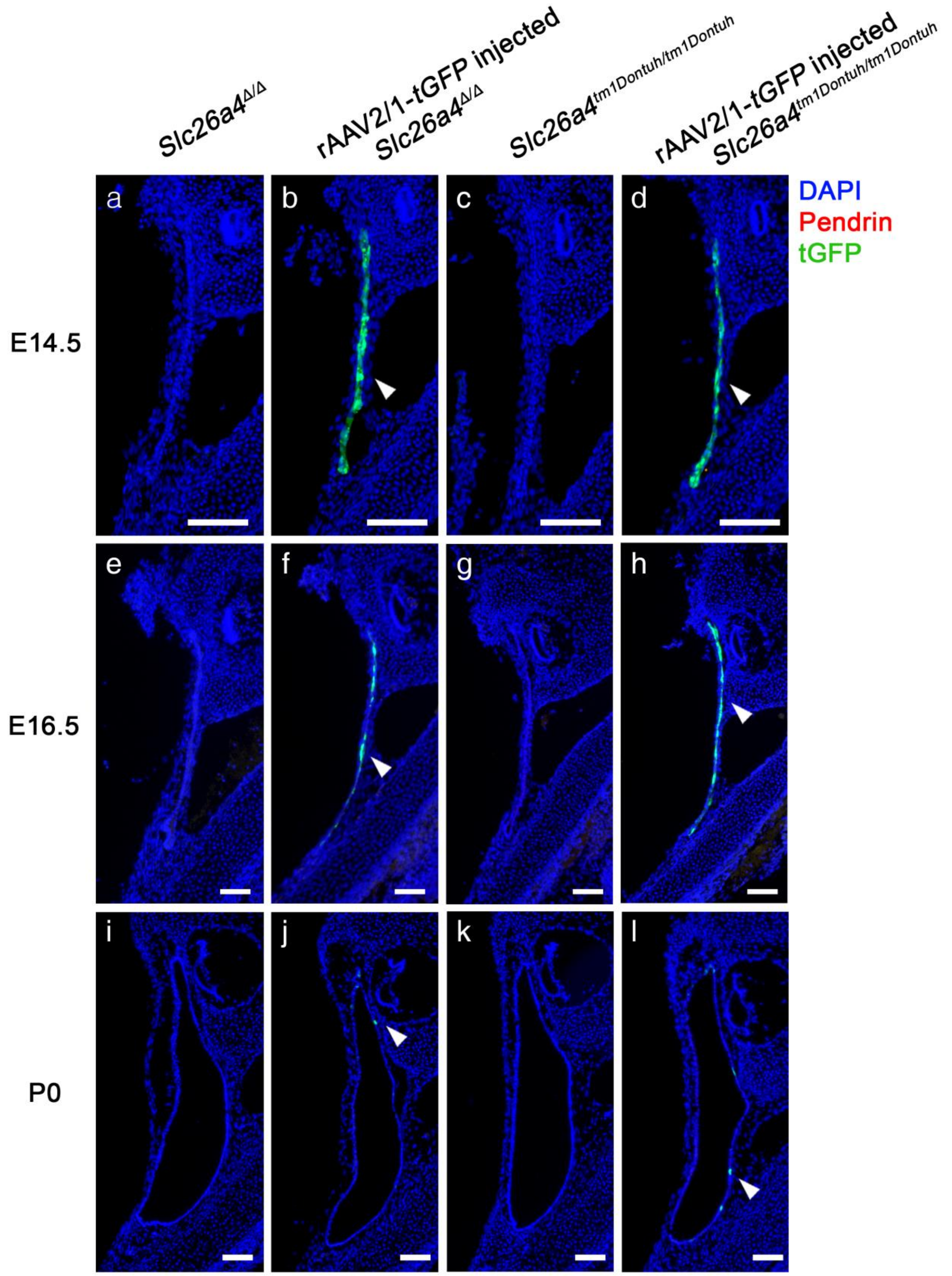

Supplementary Figure 5. **tGFP expression in the endolymphatic sac.**

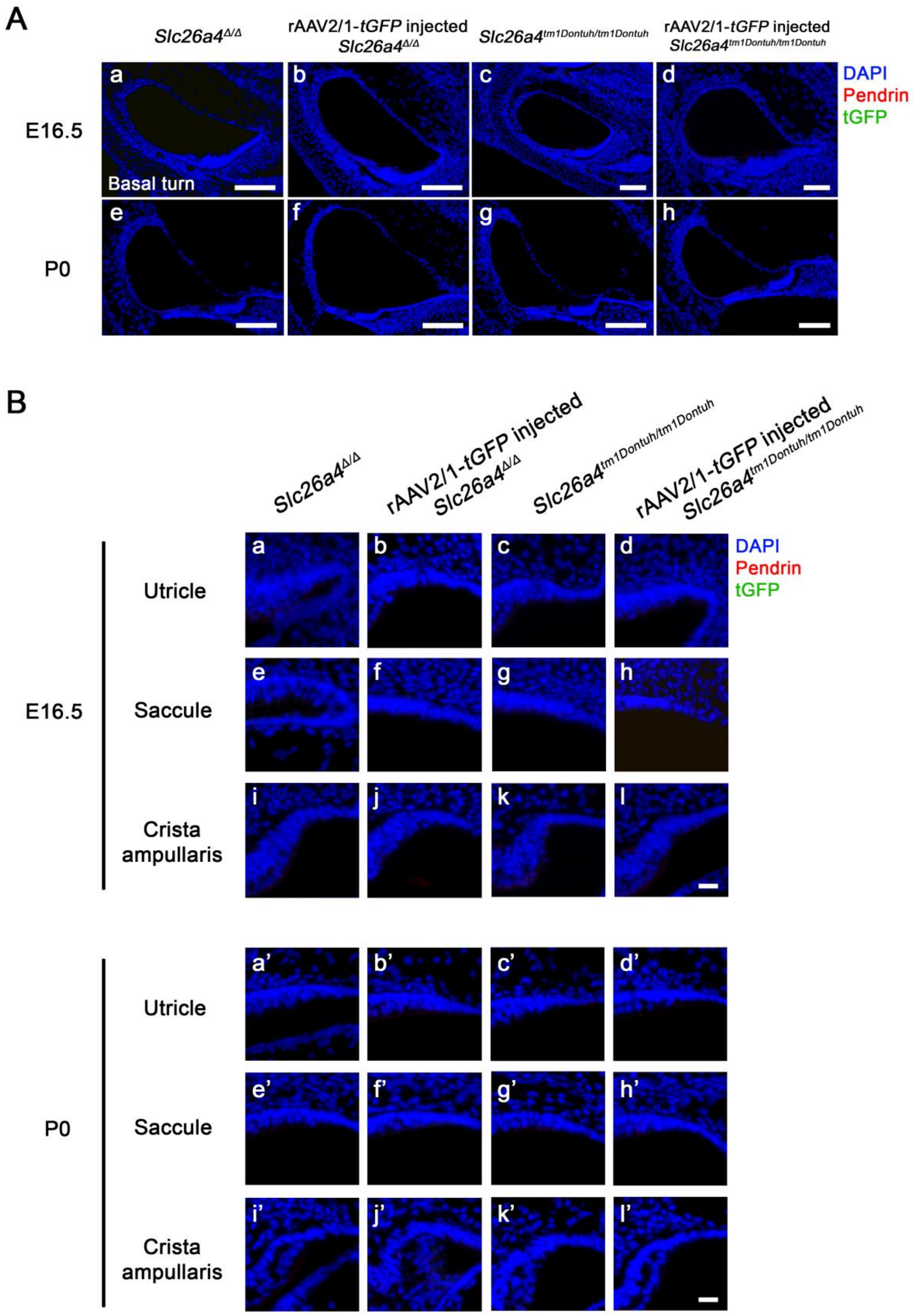

Supplementary Figure 6. **tGFP expression in the cochlea and vestibular transitional cells.**

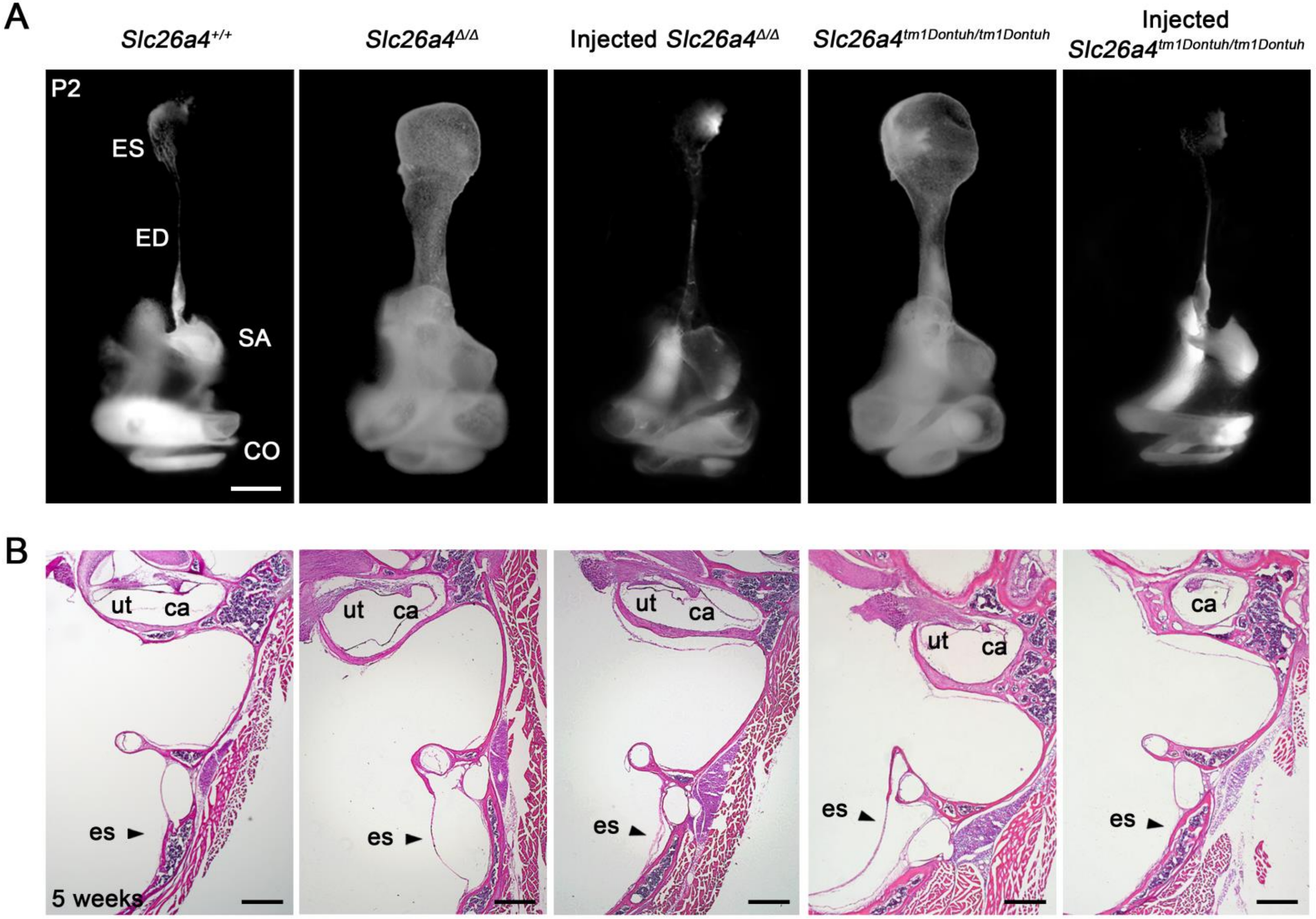

Supplementary Figure 7. Local delivery of Slc26a4 prevents enlargement of the membranous labyrinth.

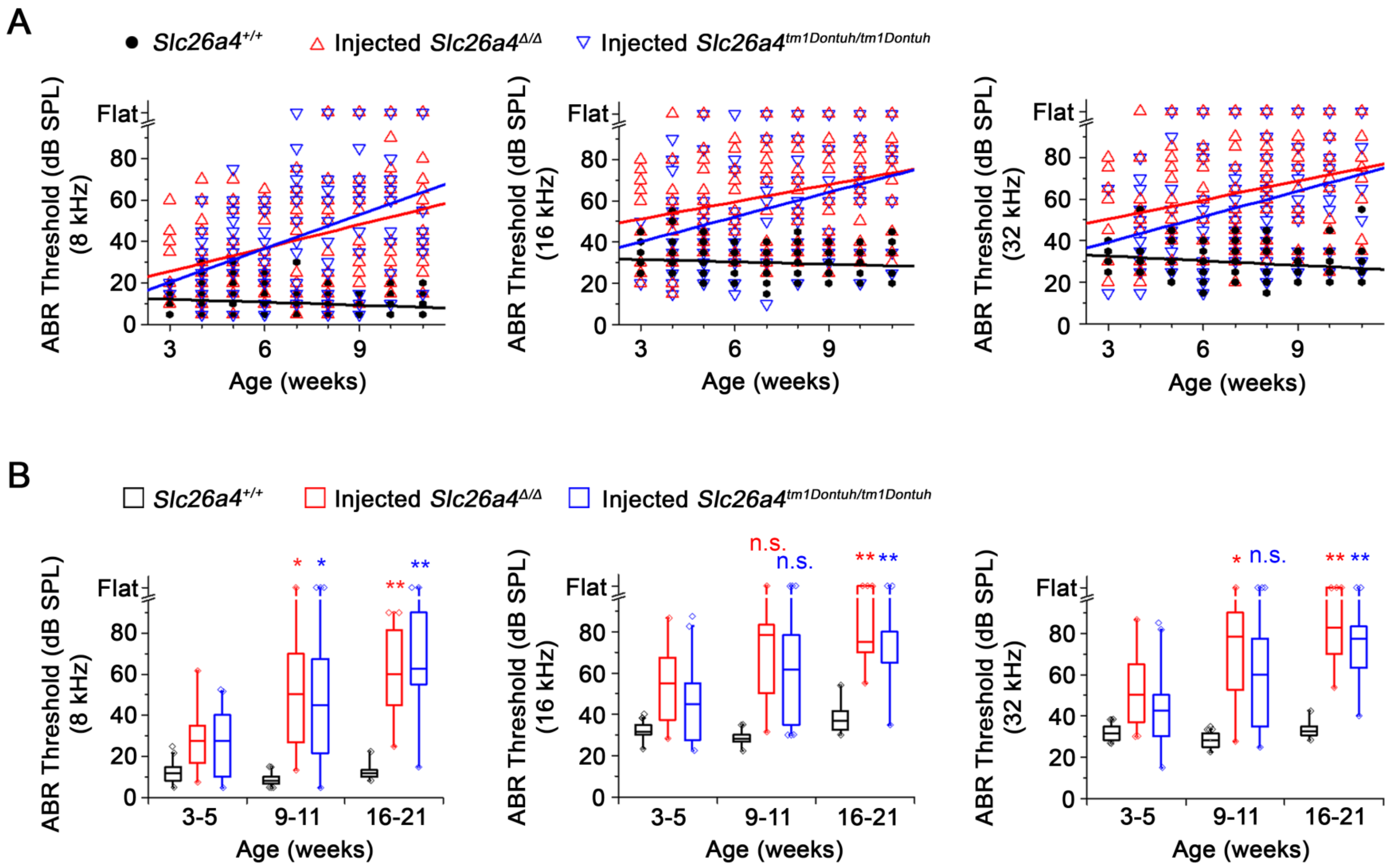

Supplementary Figure 8. **Restored hearing phenotype is unstable.**

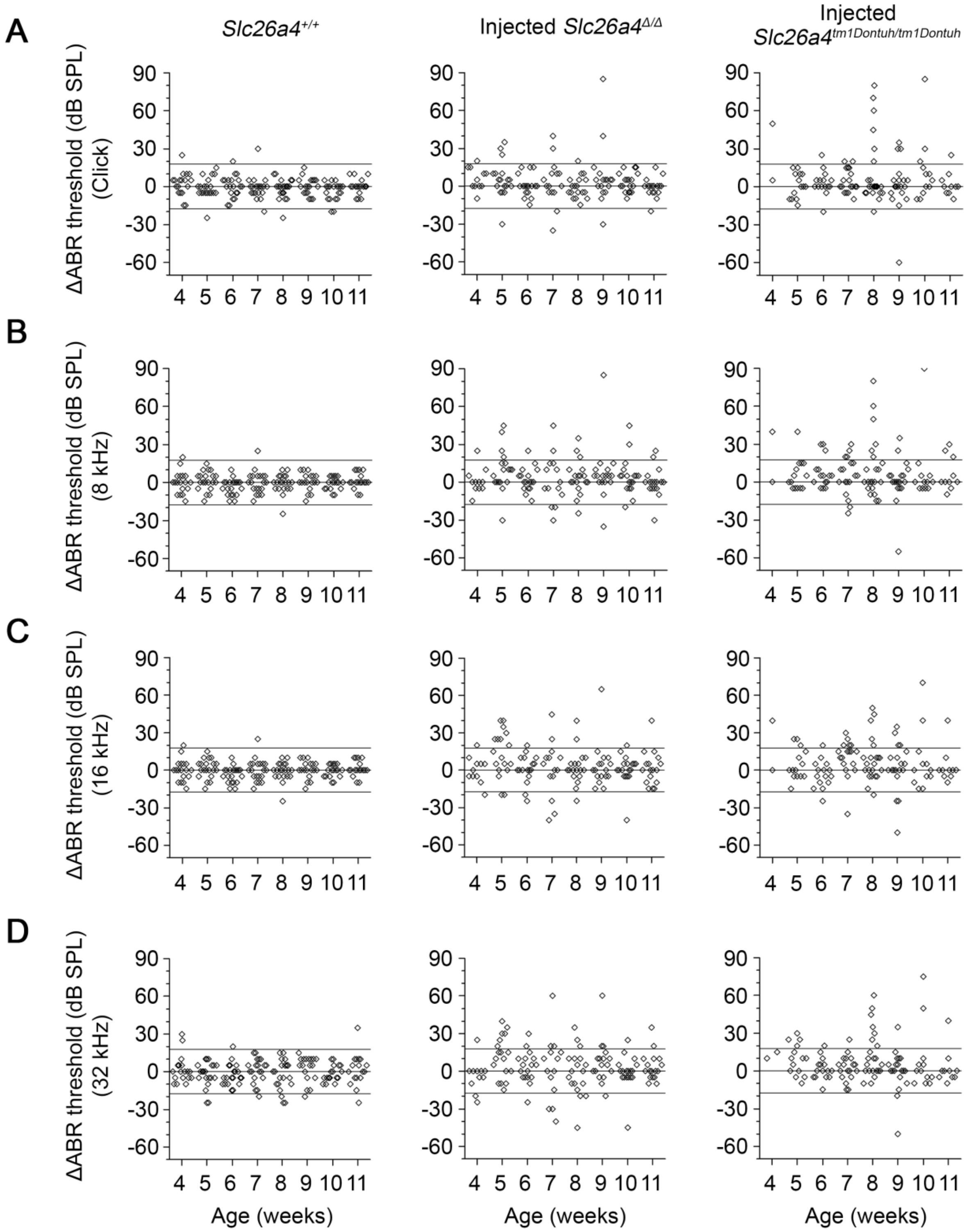

Supplementary Figure 9. **Fluctuating hearing loss.**

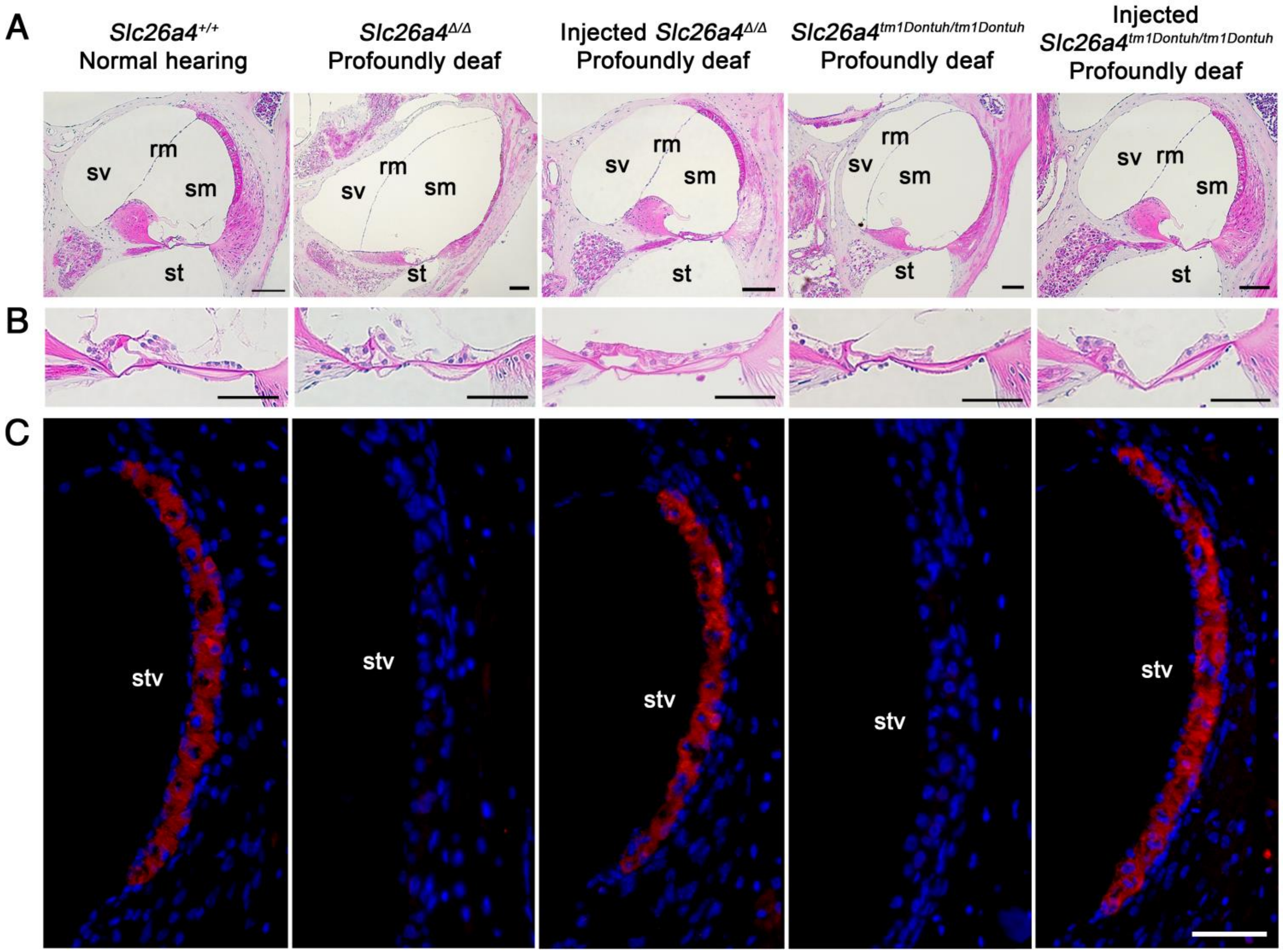

Supplementary Figure 10. **Loss of outer hair cells in profoundly deaf *Slc26a4*-deficient mice..**

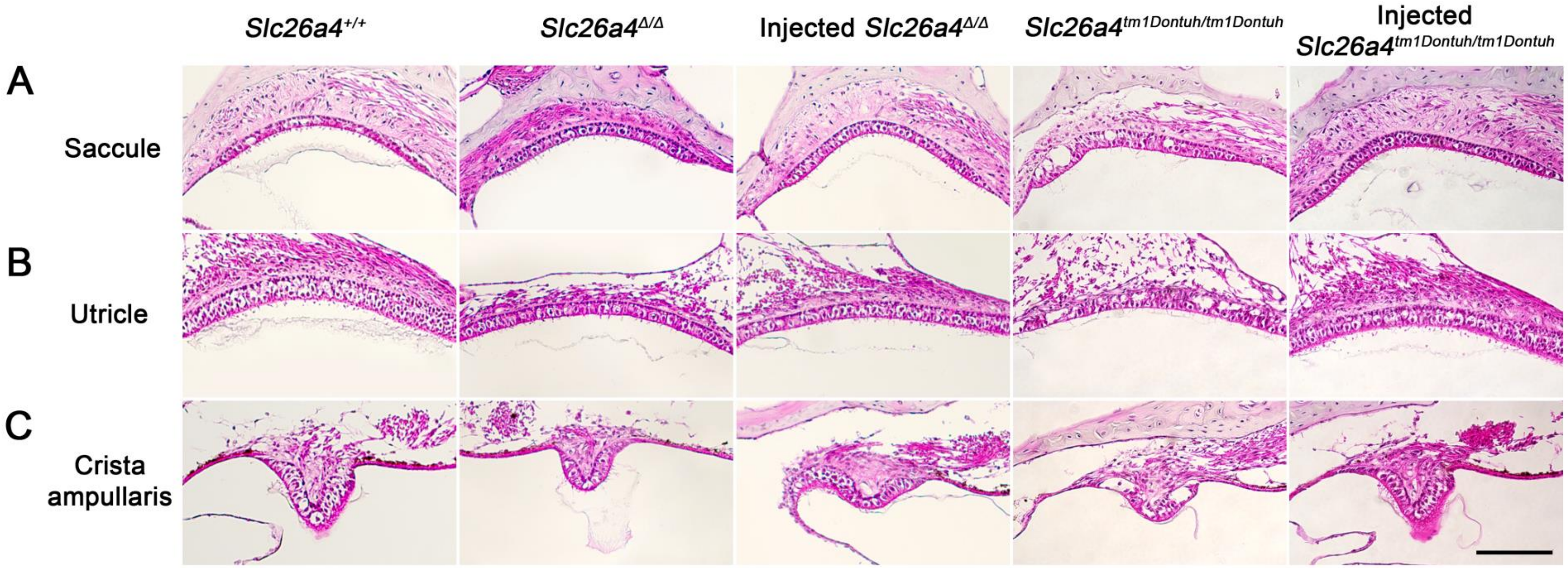

Supplementary Figure 11. **Histology of the utricle, saccule and crista ampullaris**

Supplementary figure legends

Supplementary Figure 1. **The detailed vector maps of rAAV2/1-*tGFP* and rAAV2/1-*Slc26a4-tGFP*.** (**A**) The rAAV2/1-*tGFP* vector, which was used as a control, expressed only *turbo GFP* (*tGFP*) under the control of the CMV promoter. (**B**) The rAAV2/1-*Slc26a4-tGFP* vector expressed *Slc26a4* and *tGFP* driven by the same CMV promoter. CMV, cytomegalovirus; Slc26a4, solute carrier family 26 member 4. GFP, green fluorescent protein.

Supplementary Figure 2. **Pendrin expression in the cochlea.** Pendrin immunoreactivity (*red*) and direct fluorescence of tGFP (*green*) in cross sections of the basal turn of the cochlea at E16.5 (**a-e**) and P0 (**f-j**). Endogenous pendrin expression was observed in the outer sulcus epithelium of *Slc26a4*$^{+/+}$ mice (**a, f**). No appreciable pendrin immunoreactivity or tGFP fluorescence was observed in the cochlea of *Slc26a4*$^{\Delta/\Delta}$, injected *Slc26a4*$^{\Delta/\Delta}$, *Slc26a4*$^{tm1Dontuh/tm1Dontuh}$, and injected *Slc26a4*$^{tm1Dontuh/tm1Dontuh}$ mice indicating that injection of the rAAV2/1-*Slc26a4-tGFP* vector did not induce protein expression in the cochlea. Representative images of 3 replicates each. Nuclei were stained with DAPI (*blue*). White arrowheads point to a representative pendrin-expressing cells. Scale bars: 50 µm.

Supplementary Figure 3. **Pendrin expression in vestibular transitional cells.** Pendrin immunoreactivity (*red*) and direct fluorescence of tGFP (*green*) in cross sections of the utricle (**Aa-e, Ba-e**), saccule (**Af-j, Bf-j**), and ampulla (**Ak-o, Bk-o**) at E16.5 (**A**) and P0 (**B**). Representative images of 3 replicates each. Endogenous pendrin expression was observed in vestibular transitional epithelial cells of *Slc26a4*$^{+/+}$ mice (**Aa,f,k** and **Ba,f,k**). No appreciable pendrin immunoreactivity or tGFP fluorescence was observed in transitional cells of *Slc26a4*$^{\Delta/\Delta}$, injected *Slc26a4*$^{\Delta/\Delta}$, *Slc26a4*$^{tm1Dontuh/tm1Dontuh}$, and injected *Slc26a4*$^{tm1Dontuh/tm1Dontuh}$ mice

indicating that injection of the rAAV2/1-*Slc26a4-tGFP* vector did not induce protein expression in vestibular transitional cells. Nuclei were stained with DAPI (*blue*). White arrowheads point to a representative pendrin-expressing cells. Scale bars: 50 µm.

Supplementary Figure 4. **Pendrin expression in vestibular hair cells.** Pendrin immunoreactivity (*red*) and direct tGFP fluorescence (*green*) in cross sections of the utricle (**a-c**), saccule (**d-f**), and ampulla (**g-i**) at P10 in injected *Slc26a4*$^{tm1Dontuh/tm1Dontuh}$ mice. Vector-induced pendrin and tGPF expression was observed in a subset of vestibular hair cells indicating that injection of the rAAV2/1-*Slc26a4-tGFP* vector induced ectopic expression in vestibular hair cells. Nuclei were stained with DAPI (*blue*). White arrowheads point to a representative pendrin-expressing cells. Scale bars: 50 µm.

Supplementary Figure 5. **tGFP expression in the endolymphatic sac.** Pendrin immunoreactivity (*red*) and direct fluorescence of tGFP (*green*) were evaluated in endolymphatic sacs of *Slc26a4*$^{\Delta/\Delta}$, injected *Slc26a4*$^{\Delta/\Delta}$, *Slc26a4*$^{tm1Dontuh/tm1Dontuh}$, and injected *Slc26a4*$^{tm1Dontuh/tm1Dontuh}$ mice at E14.5 (**a-d**), E16.5 (**e-h**) and P0 (**i-l**). The ears of pendrin-deficient mice showed in figure were the contralateral ears of injected pendrin-deficient mice. White arrowheads point to representative tGFP-expressing cells. Representative images of 3 replicates each. Nuclei were stained with DAPI (*blue*). Scale bars: 100 µm.

Supplementary Figure 6. **tGFP expression in the cochlea and vestibular transitional cells.** Pendrin immunoreactivity (*red*) and direct fluorescence of tGFP (*green*) in cross sections of the basal turn of the cochlea at E16.5 (**Aa-d**) and P0 (**Ae-h**) and in cross sections of the utricle (**Ba-d, Ba'-d'**), saccule (**Be-h, Be'-h'**), and ampulla (**Bi-l, Bi'-l'**) at E16.5 and P0. No appreciable pendrin immunoreactivity or tGFP fluorescence was observed in the cochlea of

Slc26a4$^{\Delta/\Delta}$, injected *Slc26a4*$^{\Delta/\Delta}$, *Slc26a4*$^{tm1Dontuh/tm1Dontuh}$, and injected *Slc26a4*$^{tm1Dontuh/tm1Dontuh}$ mice indicating that injection of the rAAV2/1-*tGFP* vector did not induce protein expression in the cochlea and vestibular transitional cells. The ears of pendrin-deficient mice showed in figure were the contralateral ears of injected pendrin-deficient mice. Representative images of 3 replicates each. Nuclei were stained with DAPI (*blue*). Scale bars: 50 µm.

Supplementary Figure 7. **Local delivery of *Slc26a4* prevents enlargement of the membranous labyrinth.** (**A**) Lateral views of paint-filled inner ears obtained from *Slc26a4*$^{+/+}$, *Slc26a4*$^{\Delta/\Delta}$, injected *Slc26a4*$^{\Delta/\Delta}$, *Slc26a4*$^{tm1Dontuh/tm1Dontuh}$, and injected *Slc26a4*$^{tm1Dontuh/tm1Dontuh}$ mice at P2. Representative images of 5, 9, 5, 8, and 5 replicates, respectively. ES, endolymphatic sac; ED, endolymphatic duct; SA, sacculus; CO, cochlea. (**B**) H&E stained sections of endolymphatic sacs and utricles obtained from *Slc26a4*$^{+/+}$, *Slc26a4*$^{\Delta/\Delta}$, injected *Slc26a4*$^{\Delta/\Delta}$, *Slc26a4*$^{tm1Dontuh/tm1Dontuh}$, and injected *Slc26a4*$^{tm1Dontuh/tm1Dontuh}$ mice at 5 weeks of age. Representative images of 3 replicates each. ut, utricle; ca, crista ampullaris; es, endolymphatic sac. Scale bars: 20 µm in (**A**) and 400 µm in (**B**).

Supplementary Figure 8. **Restored hearing phenotype is unstable.** (**A**) Linear regressions of ABR thresholds in response to 8 kHz, 16 kHz and 32 kHz sound stimuli were obtained in *Slc26a4*$^{+/+}$ mice, injected *Slc26a4*$^{\Delta/\Delta}$ and injected *Slc26a4*$^{tm1Dontuh/tm1Dontuh}$ mice between 3 and 11 weeks of age. For thresholds in response to 8 kHz sound stimuli, slopes and Pearson's R values for *Slc26a4*$^{+/+}$, injected *Slc26a4*$^{\Delta/\Delta}$ and injected *Slc26a4*$^{tm1Dontuh/tm1Dontuh}$ mice were -0.6 dB/week and R=0.20, 2.9 dB/week and R=0.40 and 4.9 dB/week and R=0.40. In response to 16 kHz stimuli, slopes and R values were -0.5 dB/week and R=0.13, 3.7 dB/week and R=0.32, and 5.4 dB/week and R=0.37 and in response to 32 kHz stimuli, slopes and R values were -0.7 dB/week and R=0.25, 3.1 dB/week and R=0.34, and 4.1 dB/week and R=0.34, respectively.

(**B**) ABR thresholds in response to 8, 16, and 32 kHz tone burst stimuli were grouped into three age ranges, 3-5 weeks, 9-11 weeks, and 16-21 weeks to evaluate the stability of hearing in *Slc26a4*$^{+/+}$, injected *Slc26a4*$^{\Delta/\Delta}$ mice and injected *Slc26a4*$^{tm1Dontuh/tm1Dontuh}$ mice. Data are represented by box-plots (25%, 50%, and 75%) with whiskers (5% and 95%). Outliers were drawn as symbols (*diamonds*). Differences toward the 3-5 week group were evaluated either by one-way ANOVA with Bonferroni t-test or by Kruskal-Wallis one-way ANOVA on ranks and Dunn's method: n.s. no significant difference, * $p<0.05$, ** $p<0.01$, *** $p<0.001$.

Supplementary Figure 9. **Fluctuating hearing loss.** ABR thresholds differences in 1-week intervals were obtained in *Slc26a4*$^{+/+}$ mice, injected *Slc26a4*$^{\Delta/\Delta}$ and injected *Slc26a4*$^{tm1Dontuh/tm1Dontuh}$ mice based on weekly ABR measurements using click sound stimuli (**A**), 8 kHz (**B**), 16 kHz (**C**) and 32 kHz (**D**) tone burst stimuli. Positive ABR threshold differences indicate hearing losses and negative threshold differences indicate hearing improvements. Threshold differences >15 dB were considered significant. Note a greater prevalence of fluctuations in injected *Slc26a4*$^{\Delta/\Delta}$ and injected *Slc26a4*$^{tm1Dontuh/tm1Dontuh}$ mice compared to *Slc26a4*$^{+/+}$ mice.

Supplementary Figure 10. **Loss of outer hair cells in profoundly deaf *Slc26a4*-deficient mice.** (**A-C**) Cross sections of the basal turn of the cochlea (**A**), the organ of Corti (**B**), and the lateral wall from normal hearing *Slc26a4*$^{+/+}$ mice, profoundly deaf *Slc26a4*$^{\Delta/\Delta}$, profoundly deaf injected *Slc26a4*$^{\Delta/\Delta}$, profoundly deaf *Slc26a4*$^{tm1Dontuh/tm1Dontuh}$, and profoundly deaf injected *Slc26a4*$^{tm1Dontuh/tm1Dontuh}$ mice. Representative images of 3, 1, 1, 3, and 3 replicates, respectively. (**A-B**) Gross morphology was evaluated in sections stained with hematoxylin-eosin. sv, scala vestibule; rm, Reissner's membrane; sm, scala media; st, scala tympani. Scale bars: 200 µm in (**A**) and 50 µm in (**B**). (**C**) KCNJ10 immunoreactivity (*red*) was evaluated

in sections stained with DAPI (*blue*). stv, stria vascularis. Scale bar: 50 μm in (**C**). Note the loss of outer hair cells in profoundly deaf injected *Slc26a4*$^{\Delta/\Delta}$ mice and profoundly deaf injected *Slc26a4*$^{tm1Dontuh/tm1Dontuh}$ mice.

Supplementary Figure 11. **Histology of the utricle, saccule and crista ampullaris.** Cross sections of the utricle (**A**), saccule (**B**) and crista ampullaris (**C**) from *Slc26a4*$^{+/+}$, *Slc26a4*$^{\Delta/\Delta}$, injected *Slc26a4*$^{\Delta/\Delta}$, *Slc26a4*$^{tm1Dontuh/tm1Dontuh}$, and injected *Slc26a4*$^{tm1Dontuh/tm1Dontuh}$ mice were obtained at 5 weeks of age and stained with hematoxylin-eosin. Representative images of 3 replicates each. sv, scala vestibule; rm, Reissner's membrane; sm, scala media; st, scala tympani. Scale bars: 100 μm. No overt differences were observed.
